# Learning to Overexert Cognitive Control in a Stroop Task

**DOI:** 10.3758/s13415-020-00845-x

**Published:** 2021-01-06

**Authors:** Laura Bustamante, Falk Lieder, Sebastian Musslick, Amitai Shenhav, Jonathan Cohen

**Affiliations:** 1 Princeton Neuroscience Institute, Princeton University, Princeton, NJ 08540 USA; 2 Max Planck Institute for Intelligent Systems, Tübingen, Germany; 3 Cognitive, Linguistic, & Psychological Science, Brown University, Providence, RI USA; 4 Carney Institute for Brain Sciences, Brown University, Providence, RI USA; 5 Department of Psychology, Princeton University, Princeton, NJ USA

**Keywords:** Cognitive control, Cognitive plasticity, Metacognitive reinforcement learning, Self-control failure

## Abstract

How do people learn when to allocate how much cognitive control to which task? According to the Learned Value of Control (LVOC) model, people learn to predict the value of alternative control allocations from features of a situation. This suggests that people may generalize the value of control learned in one situation to others with shared features, even when demands for control are different. This makes the intriguing prediction that what a person learned in one setting could cause them to misestimate the need for, and potentially overexert, control in another setting, even if this harms their performance. To test this prediction, we had participants perform a novel variant of the Stroop task in which, on each trial, they could choose to either name the color (more control-demanding) or read the word (more automatic). Only one of these tasks was rewarded each trial and could be predicted by one or more stimulus features (the color and/or word). Participants first learned colors and then words that predicted the rewarded task. Then, we tested how these learned feature associations transferred to novel stimuli with some overlapping features. The stimulus-task-reward associations were designed so that for certain combinations of stimuli, transfer of learned feature associations would incorrectly predict that more highly rewarded task would be color-naming, even though the actually rewarded task was word-reading and therefore did not require engaging control. Our results demonstrated that participants overexerted control for these stimuli, providing support for the feature-based learning mechanism described by the LVOC model.

**Supplementary Information:**

The online version contains supplementary material available at 10.3758/s13415-020-00845-x.

## Introduction

Every day, people have to make decisions about allocating cognitive control in the service of pursuing their goals (e.g., what to pay attention to, what to hold in mind, what to stop themselves from doing). How do people learn how to allocate cognitive control across the vast range of situations they encounter? One possibility is to learn the value of allocating different control signals based on features of the environment. Such a feature-based learning mechanism would allow for transfer of what was learned in one situation to other situations that share its features. For example, a driver may learn that it is valuable to attend to the speedometer, the distance to the car in front, and a navigation device in response to different features of the environment when driving. A student driver may learn what to attend by instruction or trial and error at first, but in a new situation (e.g., a different car) with shared features (e.g., a steering wheel, foot pedals), they can quickly transfer what that have learned and be able to drive effectively. However, transfer in learning is not always adaptive. For example, a person may learn that it is valuable to attend to a text message when a notification sounds. When that person is driving a car and a text notification sound appears, they may transfer what they have learned and attend to the text message, but this decision could result in a car crash. Here, we examined how examples of maladaptive control allocation can develop from otherwise adaptive mechanisms for learning the value of control.

We based our examination on a recently developed model that describes cognitive control allocation as the result of a cost-benefit analysis. Individuals weighed the expected payoffs for engaging control against the effort-related costs associated with doing so to determine the overall Expected Value of Control (EVC) for a particular control allocation (Shenhav et al., 2013; Musslick et al., 2015). Building on previous models of strategy learning (Lieder & Griffiths, 2017), we recently described a set of learning algorithms that would allow someone to learn EVC through experience performing different tasks (Lieder et al., 2018). According to this Learned Value of Control (LVOC) model, people learn to predict the value of control based on features of the task environment, and they select their control allocation accordingly. Reward that resulted from a particular control policy is compared with predicted reward to update the association of each feature with the value of control.

The LVOC model predicts that knowledge about the value of allocating control (e.g., attend to the distance of the car in front of you) will transfer to new situations that share stimulus features (e.g., other vehicles with steering wheels or foot pedals). For instance, if a given stimulus feature is associated with reward, individuals will learn to attend more to other stimuli that share this feature. Krebs et al. (2010) provided evidence in support of this, by having participants perform a classic control-demanding task, the Stroop task (Stroop, 1935), with stimulus features that predicted different levels of reward. As is typical for this task, participants were shown a series of color words (e.g.,  etc.) and asked to name the color in which the word was displayed (e.g., to respond “green” when presented with the stimulus ). It is widely assumed that this demands control to overcome interference from the more automatic tendency to read the word (i.e., respond “red ” in the previous example) (Posner & Snyder, 1975; Cohen et al., 1990). Krebs and colleagues rewarded participants monetarily after responding to words displayed in some colors (e.g., ) but not others (e.g., ). Participants came to name the color faster and more accurately for those that were rewarded than those that were not. We have shown that the LVOC model can account for such findings by decomposing a task into its component features and learning the predictive relationship between those features and future rewards (Lieder et al., 2018). For the Stroop task, each stimulus can be decomposed into two features: the color (e.g., ) and the word (e.g., **RED**). When the LVOC model was trained on Krebs et al.’s task, it learned to associate certain colors with more reward and therefore allocate more cognitive control (and thus performs better) on trials in which that color was present.

The LVOC model uses a simple linear learning algorithm that sums the predicted value of control for all features present to make its prediction. This makes learning efficient. Transfer of a high value of control often is desirable, because high amounts of control improve performance across many situations (Padmala & Pessoa, 2011, Braem et al., 2012, Krebs et al., 2010; Umemoto & Holroyd, 2015). In contrast to linear models (e.g., one layer perceptron, simple linear regression), more complex models (e.g., multilayer non-linear neural networks) can learn nonlinear contingencies (e.g., multiplicative, exclusive-OR; Rumelhart et al., 1986). However, because of their simplicity, linear models (such as the LVOC in its present form) are susceptible to certain biases in learning and may do poorly when there are nonlinearities in the value of control across different situations. Therefore, the LVOC theory predicts that the learning of the value of cognitive control can, under certain conditions, lead to “maltransfer”—that is, transfer of learning from one setting to another that turns out to be harmful or maladaptive. Krebs et al.’s experiment provides preliminary evidence in support of the hypotheses that transfer learning can hurt performance, as predicted by the LVOC theory. Even though in that experiment words were never predictive of reward, participants performed worse on incongruent stimuli that contained words for colors that were predictive of reward. For example, if the color  was associated with reward, but  was not, then the stimulus  caused more interference (maltransfer) than  (where the color  was not associated with reward). LVOC simulations suggest that this could be explained by maltransfer from experience with congruent stimuli to the incongruent stimuli. Congruent stimuli for rewarded colors ( ) decreased the learned value of control, whereas congruent stimuli for unrewarded colors ( ) did not. The LVOC-model learned that, on average, it was *less* worth allocating control when the word **GREEN** was present (compared with **RED**). When **GREEN** appeared in incongruent stimuli, this learning transferred, and participants exerted less control. Nevertheless, the study by Krebs et al. (2010) does not provide conclusive evidence for the LVOC model. First, associations between stimulus features and task goals could have been acquired through non-linear learning rather than a linear learning algorithm. Second, the LVOC suggests that maltransfer can lead to participants to overexert control, rather than underexert control as observed by Krebs and colleagues. The latter could be attributed to other factors, such as motivation, because participants tend to prefer less control-demanding tasks (Kool et al., 2010, 2013; Westbrook et al., 2013). Thus, observing overexertion would be a stronger test of the LVOC. The present study was designed to provide a more direct and rigorous test of the LVOC model.

First, we manipulated monetary reward contingencies to generate circumstances in which the LVOC model predicted that learning those contingencies would lead to overexertion of control. Second, we sought to parametrically manipulate this effect by varying the experience that participants had with different features and reward for control. Finally, rather than inferring how much control participants chose to allocate based on their response times, on each trial we required them to choose which of two tasks to perform—one that required more control (color naming [CN]) or one that required less (word reading [WR]). To implement this design, we modified the Stroop paradigm used by Krebs and colleagues by varying whether reward was associated with a higher level of control (naming the stimulus color) or lower level of control (reading the stimulus word). Thus, on every trial of the experiment, participants freely chose which task to perform (CN or WR), but only one of these tasks was rewarded. Which task would be rewarded could always be predicted by a feature of the stimulus. They were initially trained on one set of features (e.g., the color  and the word **RED**) that predicted CN would be rewarded, and another that predicted WR would be rewarded. We then presented participants with novel combinations of those learned features (in the “Transfer Phase”) and tested their ability to learn the rewarded response to those novel stimuli. Specifically, we introduced new stimuli (e.g., ) that combined features that were each previously associated with the value of exerting greater control (CN) but together now were associated with reward for performing the *less* control-demanding task (WR). The disjunctive relationship between the two features in this condition—predicting that CN is rewarded when one feature is present but not when both are (i.e., the exclusive-or rule [XOR])—renders this condition unlearnable by linear learning algorithms, such as the LVOC-driven agent. This leads to the counterintuitive prediction that participants should err in this condition by exerting too much control (i.e., choosing to engage in CN despite WR being more rewarding). Our findings confirmed this prediction. Using a between-subjects design, we demonstrated that the severity of maltransfer was experience-dependent. Across three groups of participants, we parametrically varied the frequency of trials that associated either the color or the word with CN (e.g., ). We predicted that the increased strength of the association between those features and reward for CN would increase maltransfer to the stimuli that combined the features (e.g., ). As predicted, participants exposed more to the CN associated features experienced more maltransfer and earned fewer rewards. We constructed our stimulus set with six trial types that had differential predictions for transfer. Maltransfer occurred in certain circumstances (trial types with shared features) but not others (nonoverlapping features). Our findings suggest that, as proposed by the LVOC theory, people learn the value of control as they do other simple associations and use these associations to determine how to allocate control.

## Methods

### LVOC Model

According to the LVOC model, people learn to predict the expected value of control, EVC(*s*, *c*), for a specified control signal *c* in situation *s* from the stimulus features in f(s,*c*), that is:

$$ EVC\left(s,c\right)\approx LVOC\left(s,c;w\right)=\sum \limits_i{w}_i\cdotp {f}_i\left(s,c\right)- cost\left(s,c\right), $$

where *w*_*i*_ is the weight of the *i*^*th*^ stimulus feature and *cost*(*s*, *c*) is the cost of exerting the control specified by the control signal c. As an illustration, consider a Stroop task involving stimuli composed from the colors , the words **RED** and **GREEN**, and in which exerting control results in a CN response and not exerting control results in a WR response. In this example, the features would include *f*_1_(*s*, *c*) = *colorIsBlue*(*s*, *c*) · *c*, *f*_2_(*s*, *c*) = *colorIsGreen*(*s*, *c*) · *c*, *f*_3_(*s*, *c*) = *wordIsRed*(*s*, *c*) · *c*, *f*_4_(*s*, *c*) = *wordIsGreen*(*s*, *c*) · *c*, as well as all combinations of these features (e.g., *f*_5_(*s*, *c*) = *colorIsRed*(*s*, *c*) · *wordIsGreen*(*s*, *c*) · *c*) for which each feature takes the value of the control signal intensity c if the stimulus has its preferred property (e.g., being the color , or being the combination of the color  and the word **GREEN**) and 0 otherwise. Including the control signal intensity as an additional feature (*f*_6_(*s*, *c*) = *c*) allows the model to learn about the stimulus-independent (global) value of control. The weights *w* = (*w*_1_, …, *w*_6_) of these features are learned by Bayesian linear regression. The experienced value of control (i.e., *R*_*t*_ − *cost*(*s*, *c*_*t*_)) is regressed onto the features *f*, where *R*_*t*_ is the reward experienced upon control allocation and *cost*(*s*, *c*_*t*_) is the disutility of allocating the control signal. The cost includes a response time cost, an implementation cost that scales with the amount of control allocated, and a reconfiguration cost that penalizes diverging from the most recent control signal, that is

$$ cost\left(s,{c}_t\right)=\underset{response\ time\ cost}{\underbrace{\omega \cdotp RT}}+\underset{implementation\ cost}{\underbrace{\mathit{\exp}\left(\alpha +\beta \cdotp |{c}_t|\right)}}+\underset{reconfiguration\ cost}{\underbrace{\mathit{\exp}\left(\alpha +\beta \cdotp |{c}_t-{c}_{t-1}|\right)}} $$

with control cost parameters set to *α* =  − 1 and $$ \beta =\frac{1}{4} $$. The chosen parameters express the assumption that the control cost increases with the control intensity at a moderate rate. We set the cost parameter alpha to −1 to ensure that, if no control is applied (*c*_*t*_ = 0), then the control cost evaluates to 0. Beyond this, the model is robust to the exact values of the control cost parameters, as are the resulting predictions. Following Lieder et al. (2018), the opportunity cost parameter was set to *ω* = 0.44 points per second, which corresponds to an hourly wage of about $8/hour. The prior distribution on each weight is $$ \mathcal{N}\left(\mu, {\sigma}^2\right) $$ where *μ* and *σ* are free parameters that are shared across all weights. The resulting posterior distribution *P*(***w***| *E*) over the weights given the agent's experience *E* is then used to select the control signal *c*^⋆^ via Thompson sampling, that is

$$ {c}^{\star }=\mathit{\arg}\underset{c}{\mathit{\max}}\mathrm{LVOC}\left(s,c;\tilde{w}\right), where\tilde{w}\sim P\left(\boldsymbol{w}|E\right). $$

Following Musslick et al. (2015), the LVOC model translates control signals into response times and error rates via a drift-diffusion model with the drift rate:

$$ d={c}^{\star}\cdotp {y}_{\mathrm{color}}\cdotp {d}_{controlled}+\left(1-{c}^{\star}\right)\cdotp {y}_{word}\cdotp {d}_{automatic} $$

where *y*_*color*_, *y*_*word*_ ∈ {−1, 1} are the responses associated with the color or word, respectively, and *d*_*controlled*_ and *d*_*automatic*_ are the drift rates of the automatic (WR) process and the controlled (CN) process, respectively. We simulated the DDM to yield a response and response time on each trial. If the response was consistent with the rewarded task (CN or WR) for a trial type, the model received a reward of a particular number of points (see below). Otherwise, the model received no reward. In both cases, the model agent is penalized for its response time and the other cognitive control costs based on how much control it allocated (in the experiment described below, although participants were not explicitly penalized for longer response times, this did diminish their overall reward rate). The model learns from the difference between the reward and the penalties as described above. We designed an experiment that varied the reward for controlled responses to test the core predictions of the LVOC model; that learning of the value of control is based on features and transfers across situations that share features.

### Experiment

#### Experiment design

We tested predictions of the LVOC model, and in particular maltransfer of the learned value of cognitive control, by manipulating reward associated with allocating control to incongruent Stroop stimuli. Participants were tasked with learning which stimulus features predicted that CN would be rewarded (control-demanding response) and which predicted that WR would be rewarded (automatic response). There were two phases of the experiment. In an initial “Mapping Phase,” participants learned the associations between stimulus features and rewarded task. We were particularly interested in the extent to which participants learned which colors and words predicted that CN would be rewarded—that is, the value of allocating control to execute the control-demanding response—since we assumed that this involved greater effort (i.e., was associated with a greater cost of control) and therefore would only be performed when the participant predicted that it would be rewarded (otherwise, they should prefer WR as the less effortful, automatic response). Next, in the “Transfer Phase,” we presented stimuli containing novel combinations of the features used in the Mapping Phase (see Table 2 for stimulus set). We predicted participants would transfer what they learned about the value of control for individual stimulus features in the Mapping Phase to the novel combinations of those features in the Transfer Phase. We were particularly interested in feature combinations for which the LVOC model predicts maltransfer; these were combinations in which the individual features both predicted reward for CN in the Mapping Phase, but their combination predicted WR in the Transfer Phase. For example, if in the Mapping Phase the color  and the word **RED** each predicted that CN would be rewarded (and neither of which appeared with the other), then, in the Transfer phase, we presented a new stimulus that combined these features (i.e., ) but that predicted WR would be rewarded (corresponding to an XOR rule for the two features). The LVOC model predicted that participants should perform poorly on these stimuli, choosing CN over WR. This is because they should experience transfer of the associations previously learned for each feature individually (i.e., that it predicted CN would be rewarded) to the new stimulus that contained them both. Our key prediction of maltransfer was tested at the end of experiment, during which participants might have been less engaged (Randles et al., 2017). To ensure that participants were motivated to make goal-consistent responses, we doubled the reward from 5 points in the Mapping Phase to 10 points in the Transfer Phase. We hoped this manipulation would mitigate effects of fatigue and/or boredom on performance. In support of this, we observed that, whereas participants showed the predicted maltransfer, their performance on BOTH trials improved over the course of the Transfer Phase, indicating sustained engagement (see Results section, Figure 5). Furthermore, we would predict that fatigue would lead to more WR responses and therefore would not explain observed maltransfer.

We named trial types in the Transfer Phase according to their relationship to the XOR rule (Table 1). Each individual feature in the experiment was either “CN-mapped” (predicted CN was rewarded) or not. Whether the CN response was rewarded for a stimulus was determined by how many of maximally two CN-mapped features were present (neither, either, or both). If, during the Transfer Phase, the stimulus contained no features that had been CN-mapped during the Mapping Phase (“NEITHER” trial type), then CN was not rewarded (and WR was). This condition only occurred in the Transfer Phase. If the stimulus contained *only one* CN-mapped feature (“EITHER-COLOR” and “EITHER-WORD,” collectively referred to as “EITHER” trial type), then CN was rewarded as it had been during the Mapping Phase. Finally, if the stimulus contained both CN-mapped features (“BOTH” trial type), then CN was *not* rewarded (i.e., WR was rewarded instead); like NEITHER trials, this only occurred in the Transfer Phase. Features that were not CN-mapped were either WR-mapped or not mapped (control features). Control features in the Mapping Phase paired equally often with CN-mapped and WR-mapped features. Thus, control features were not relevant to the XOR rule, because they did not overlap with the CN-mapped or WR-mapped feature sets and were equally associated with a reward for CN or WR. “WR CONTROL trials” served as a matched control for the LVOC model's prediction regarding maltransfer on “BOTH” trials. This is because WR was the rewarded task in both cases, but the linear learning rule predicted maltransfer only for the BOTH trials. Therefore, WR CONTROL trials provided a benchmark for overall preferences for CN versus WR. Finally, “CN CONTROL trials” were included so that CN was rewarded for exactly half of Transfer Phase trials.

**Table 1.** Summary of trial types. Shows how trial types in Transfer Phase were related to the mapping of their stimulus features during the Mapping Phase. For example, in the second row (EITHER-COLOR trials), the color feature was rewarded for CN in the Mapping Phase (first column), the word feature was rewarded for CN in the Mapping Phase (second column), and the combination of those features was rewarded for the CN response (third column). For CONTROL trials (last two rows), the color and word features were equally often rewarded for CN and WR in the mapping phase. Dash indicates that none of the features of that trial type were rewarded for that task in the Mapping Phase.

Trial type	Stimulus features rewarded for CN in Mapping Phase	Stimulus features rewarded for WR in Mapping Phase	Rewarded response at Transfer Phase
NEITHER	-	Color & word	WR
EITHER-COLOR	Color	Word	CN
EITHER-WORD	Word	Color	CN
BOTH	Color & word	-	WR
WR CONTROL	Equal experience for color & word	WR	WR
CN CONTROL	Equal experience for color & word	CN	CN

Within-participants, we measured the proportion of CN responses in BOTH trials compared with WR CONTROL trials (i.e., for which no task had been reinforced for the component features and WR also was the rewarded response). The extent to which each participant was more likely to choose CN on BOTH trials than WR CONTROL trials was taken as evidence of maltransfer due to prior experience with the component features of these stimuli.

We also used a between-participants manipulation to test for a parametric effect of maltransfer to BOTH trials. We did this by manipulating the frequency of EITHER trials (e.g., ) in the Transfer Phase across three groups of participants and then comparing the proportion of CN responses to BOTH trials (e.g., ) observed in each group. The rationale for this was as follows: CN was the rewarded response for EITHER trials, which shared a feature with BOTH trials (e.g., color  or the word **RED**). Therefore, reinforcement from EITHER trials should increase the value of control for their features, and this value of control should increase the likelihood of exerting control and CN on BOTH trials. The frequency of EITHER trials was either 0%, 20%, or 50% of Transfer Phase trials in the three groups. The extent to which participants were more likely to CN in higher EITHER frequency groups was taken as evidence for maltransfer being graded, experience-dependent, and due to a linear learning algorithm.

#### Task design

**Figure 1.**
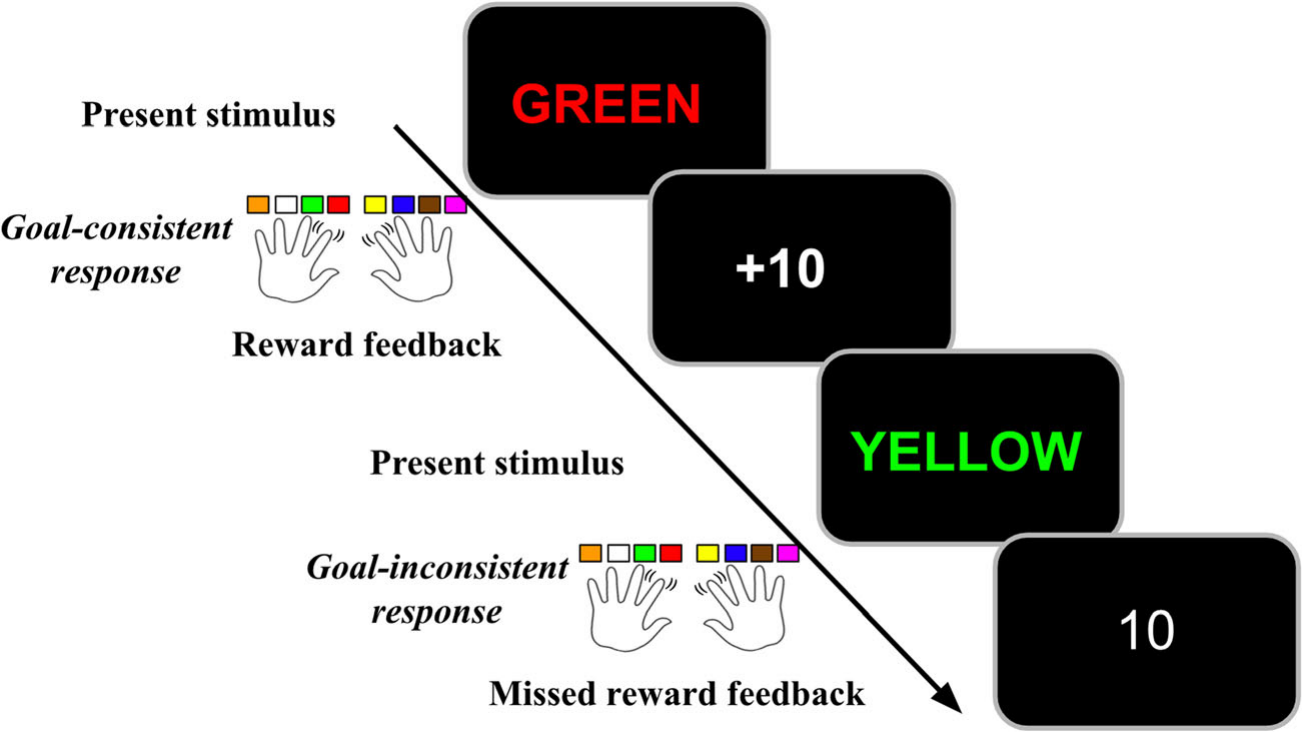
Trial structure. Participants were tasked with either responding to the color or word of a Stroop stimulus. The stimulus remained on screen until they responded. Following a response, reward feedback was displayed. After feedback, the next trial began immediately.

Participants were instructed that they would see color words displayed in a colored font and that they must respond by either pressing the key associated with the color of the font (CN) or the word (WR). Participants were told they could perform either the WR or CN task on any trial. There were no congruent trials, and we assumed that if participants gave a response that was correct for either of the two tasks, that was the task they chose to perform, and they were rewarded accordingly. The two tasks were differentially rewarded according to the particular features of the stimulus (Table 2). The participants were never instructed on the relationships of the features to the relative reward of the two tasks; they had to learn that by experience (for exact instructions see Supplementary). On each trial, the stimulus appeared and remained on screen until the participant responded. After a response, the trial ended and feedback showing the amount of reward received was displayed (Figure 1). Participants were instructed that they had a maximum of 3 seconds to respond. Within this window trials were self-paced, so that faster response times increased reward rate and decreased total time in the experiment. Following a rewarded response, participants saw the number of points they earned in bold font with a plus sign (e.g., **+10**). Following an unrewarded response they saw how much reward they missed in regular font without a plus sign (e.g., 10). Feedback was displayed on the screen for 1 second, and there was a brief 100-ms intertrial interval consisting of a blank screen before the next stimulus was presented.

**Table 2.**
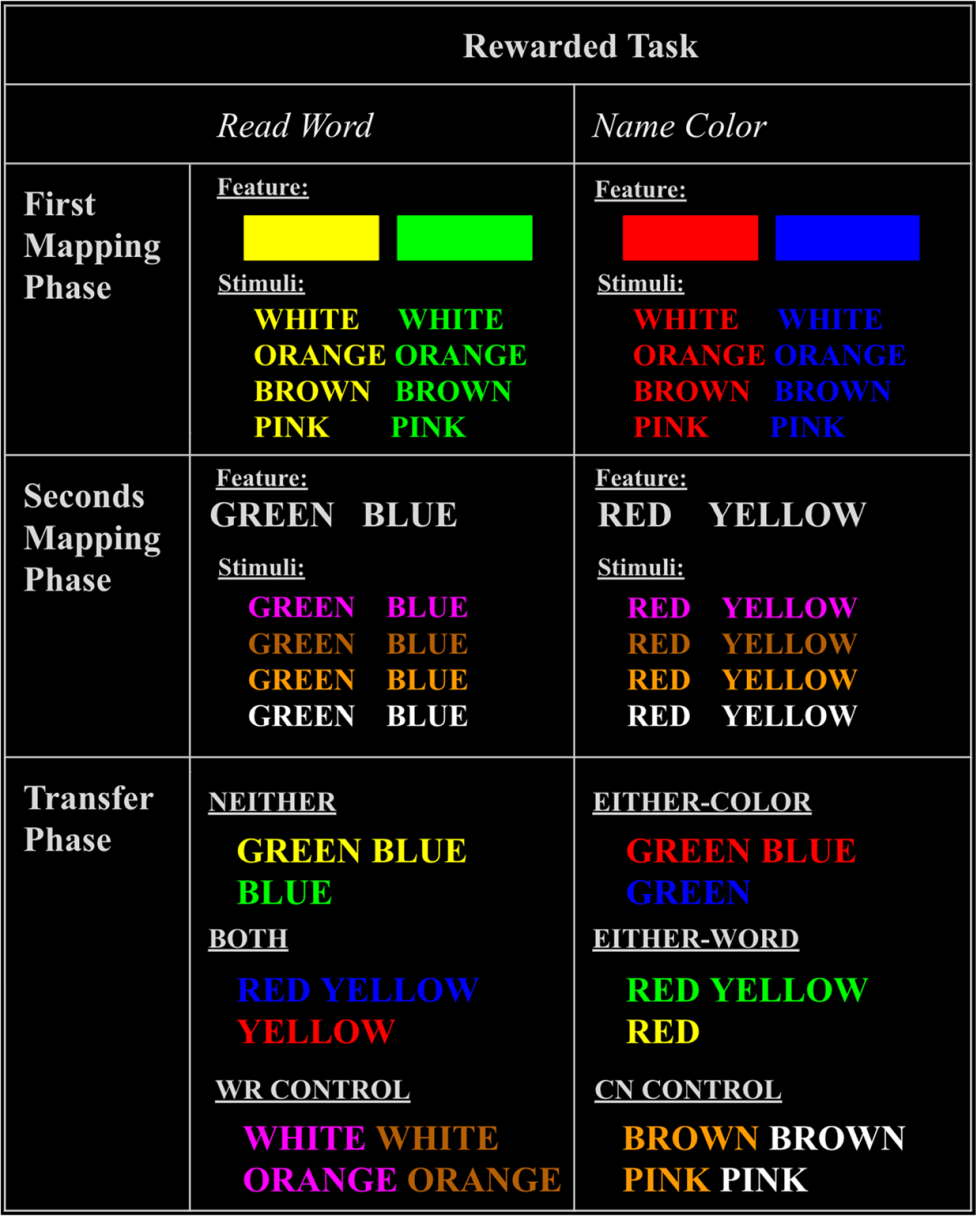
Experiment stimuli. Columns are organized by rewarded task. First column: trials for which WR was rewarded; second column: trials for which CN was rewarded. Rows are organized by experiment phase. First row: Mapping Phase Part 1, in which color features of interest were trained in combination with control words; second row: Mapping Phase Part 2, in which word features of interest were trained in combination with control colors; third row: Transfer Phase stimuli, in which trial type names refer to the designation of the combined features in the XOR rule.

Participants completed trials in two sequential phases: the Mapping Phase and the Transfer Phase (see Table 2 for full stimulus set). The Mapping Phase consisted of two parts, in which participants were trained on stimulus-task mappings for “features of experimental interest” (yellow, green, red, blue), first for colors and then for words. Features of experimental interest were used to test effects of transfer to the new stimuli in the Transfer Phase. Thus, in the first part of the Mapping Phase, stimulus-task associations were based exclusively on the color feature of the stimuli, using all of the color features of experimental interest ( ). For example, every time the stimulus was displayed in the color , the CN task was rewarded, and every time it was in the color , the WR task was rewarded. Again, participants were not explicitly instructed about these associations; they had to be learned by trial and error. In the second part of the Mapping Phase, stimulus-task associations were based exclusively on the word feature of the stimuli, for all of the word features of experimental interest (**YELLOW**, **GREEN**, **RED**, **BLUE**). For example, every time the word **RED** was presented, the CN response was rewarded, and every time the word **GREEN** was presented, the WR response was rewarded. In the Mapping Phase, the features of interest were combined with “control features” (white, orange, brown, pink) for which no specific feature-task mappings were trained in the Mapping Phase.

In the Transfer Phase, we examined participants’ propensity to allocate cognitive control to new stimuli (i.e., combinations of colors and words not presented during the Mapping Phase). There were six trial types, four of which (NEITHER, EITHER-COLOR, EITHER-WORD, and BOTH) involved combinations of features of experimental interest (i.e., ones that had individually established feature-task associations, but had not been seen together, in the Mapping Phase), and the other two of which (WR CONTROL and CN CONTROL) combined control features that had been rewarded equally often for CN as WR in the Mapping Phase (see Table 1 for a summary of the trial types). The control trial types had no features in common with each other and allowed us to isolate effects on performance that were specific to feature-based transfer.

#### Between-participants manipulation of trial type frequency

We sought to test for a parametric effect of maltransfer to BOTH trials by manipulating the frequency of CN-mapped features in the Transfer Phase (EITHER trials). We predicted that increased experience with EITHER trials would increase maltransfer to BOTH trials on the basis of their shared features (i.e., greater exposure to the EITHER conditions would worsen performance in the BOTH condition). EITHER trials were 0%, 20%, or 50% of Transfer Phase trials in the three EITHER frequency groups (see Table 3 for frequencies of all trial types by EITHER frequency group). The set of stimuli in the Mapping Phase were the same for all groups, as was the frequency of BOTH trials (20%) and NEITHER trials (10%).

**Table 3.** Between participants frequency manipulation of EITHER trials. The three experimental conditions (columns) differ in the relative frequencies of the different trial types (rows) in the transfer phase. Rows are trial types. Columns are between-participant groups.

Trial Type	Experimental condition		
	0% EITHER TRIALS	20% EITHER TRIALS	50% EITHER TRIALS
**EITHER**	**0%**	**20%**	**50%**
CN CONTROL	50%	30%	0%
**BOTH**	**20%**	**20%**	**20%**
WR CONTROL	20%	20%	20%
NEITHER	10%	10%	10%

The LVOC model also predicted increased CN for NEITHER trials (e.g., ). This was because these trials shared the WR-mapped feature of EITHER trials (e.g., **GREEN** and ). The LVOC model predicts that greater exposure to EITHER trials reinforces the association between the WR-mapped feature in these trials and CN. This leads to maltransfer when the same WR-mapped feature is present in NEITHER trials. However, maltransfer to NEITHER trials was predicted to be to a lesser degree than the maltransfer to BOTH trials, because there was no interference from Mapping Phase training to NEITHER trials.

The control trial types were used to ensure that manipulating the frequency of EITHER trials across groups (in order to test its effect on maltransfer) was not confounded with an overall increase in the likelihood of reward for CN across groups. Specifically, the frequency of CN CONTROL trials was adjusted so that the *overall* likelihood of reward for CN was maintained at 50% of Transfer Phase trials for all groups. Thus, CN CONTROL trials were made more frequent for groups in which EITHER trials were less frequent, and vice versa.

WR CONTROL trials served to measure any changes in the global value that may have occurred across EITHER frequency groups. WR CONTROL trials were frequency matched to BOTH trials and both were rewarded for WR. The key difference is that unlike BOTH trials, WR CONTROL trials (for example, ) did not share color and word features with EITHER trials (for example, ). Therefore, responses to WR CONTROL trials should not change across EITHER frequency groups on the basis of their color and word features. WR CONTROL trials served as a baseline against which to compare BOTH trials. On the one hand, a greater tendency to CN on BOTH compared with WR CONTROL would reflect feature-specific transfer effects. Conversely, if responses on WR CONTROL trials did change across EITHER frequency groups, this would suggest a change in the learned global value of control.

#### Block design

Participants completed 520 total trials: 160 trials in each of the two parts of the Mapping Phase, and 200 trials in the Transfer Phase. During the Mapping Phase, participants were rewarded 5 points for each rewarded response. During the Transfer Phase, we doubled the reward to 10 points per rewarded response in an effort to counteract the potential contribution of waning motivation to maltransfer in the final phase of the experiment. At the end of each part of the Mapping Phase, participants had a self-paced break and saw how many points they earned in that Phase. Additionally, participants had a self-paced break in the middle of the Transfer Phase and saw how many points they earned at the end of the Transfer Phase.

#### Counter-balancing

Participants were assigned to one of the three EITHER frequency conditions in a counterbalanced order. For a given trial type (e.g., BOTH) there were several unique possible stimuli (e.g., ). The presentations of unique stimuli were balanced within the total number of trials for a given trial type. The number trials of each trial type was fixed for a given EITHER frequency condition (according to the frequencies in Table 3). Trial order was randomized within each phase.

#### Keypress training

We used eight colors and eight words (yellow, green, red, blue, white, orange, brown, pink). The keyboard was equipped with colored stickers to aid participants in remembering which color corresponded to which key throughout the experiment. Participants learned to associate the keypress with each color at the start of the experiment. During keypress training participants, were shown a nonword in color (i.e. ) and responded using either their left or right hand, and any finger besides their thumbs. Once they responded they got feedback, saying “correct” for 650 milliseconds (ms) if they were correct or else saying which key they should have pressed for 1,000 ms if they were incorrect. Participants completed 80 trials of keypress training. If they pressed the wrong key on greater than 20% of all 80 trials, they had to repeat the practice. After keypress training, participants began the main task.

### Participants

Participants were recruited from a pool administered by the Department of Psychology at Princeton University. Potential participants who were color-blind were not invited to participate in the study. Thirty adults participated in the experiment (10 per EITHER frequency condition). The demographics form was not completed by three participants, because the experiment ran over time. Of the 27 respondents, 16 participants were female, 18-53 years old (mean *[M]* = 22, standard deviation *[SD]* = 8). Demographics were similar across EITHER frequency groups (in the 0% group, 4 participants were female, *M* = 23.11 years, *SD* = 11.11; 20% group 6 participants were female, *M* = 20.77 years, *SD* = 0.97; 50% group 6 participants were female, *M* = 21.11, *SD* = 6.41). Of note, cognitive control decision-making is demonstrated to change across the lifespan (Westbrook et al., 2013), so future studies may elect to use a more age homogeneous sample. All participants provided written informed consent in accordance with the Princeton University Institutional Review Board. Participants received 12 U.S. dollars for 1 hour of participation as well as bonus compensation for points they earned in the task. Participants received 1 dollar of bonus compensation for every 200 points (bonus compensation ranged from 10 to 17 U.S. dollars, *M* = 13, *SD* = 1.80). By comparison, the Krebs et al. (2010) study gave 10 cents per accurate trial; we gave 10 points (5 cents) per rewarded trial. Unlike the Krebs et al. (2010) study, we did not include penalties (lose 10 cents for error). Both studies gave feedback on total earnings throughout the task during breaks (both studies had 4 breaks), and the total bonus was similar (average $15 in their task, $13 in this task).

### Analysis

#### Model-free analysis of behavior

We tested the hypothesized effects of the EITHER trial frequency manipulation on responses for each of the Transfer Phase trial types using regression analyses of responses and response times, as well as drift diffusion modeling. When participants responded to the feature corresponding to the rewarded task, we refer to a “goal-consistent response” (e.g., the color feature for a trial type rewarded for CN). When participants responded to the feature corresponding to the unrewarded task (e.g., the word feature for a trial type rewarded for CN) we refer to a “goal-inconsistent” response, on the assumption that participants’ had the rational goal of maximizing their reward. We tested the effect of EITHER frequency group on the probability of goal-inconsistent responses by fitting mixed-effects logistic regression models (LMER) separately for each of the Transfer Phase trial types using the lme4 package (Bates, Maechler, Bolker, & Walker, 2015) in the R statistical language (http://www.r-project.org/). The parametric manipulation of EITHER frequency across groups was treated as a continuous variable. Participants were treated as random effects, in which estimates of each participant’s probability of goal-inconsistent responses were distributed around a group mean estimate. We also tested for an effect of an overall decrease in reward rate in higher EITHER frequency groups. To do so, we computed participants’ reward rate in the Transfer Phase as the sum of rewards divided by the total time on task (not including breaks) in the Transfer Phase (which varied by participants given their response times) and linearly regressed the reward rate against EITHER frequency group. Finally, we performed pairwise comparisons of certain trial types for which the LVOC model made specific predictions. To test whether two trial types had different effects on behavior, we regressed trial type (using only data for the trial types being compared) and the EITHER frequency condition onto the probability of goal-inconsistent responses using mixed-effects logistic regression. We tested whether participants improved on BOTH trials over the course of the Transfer Phase. To do so, we applied logistic mixed-effects regression and predicted goal inconsistent responses for BOTH trials with fixed effects of an intercept, EITHER trial frequency group and BOTH trial number, and random effects of intercept and BOTH trial number. Analyses of response times are available in the supplementary information.

The present study was limited to 30 participants, with repeated-measures within participants (840 trials each). We conducted a Bayesian analysis to quantify exactly how confident we can be about each of the positive and negative findings. Specifically, we computed the Bayes Factor for each test, which is able to (1) distinguish between inconclusive results and null results, and (2) quantify evidence for the null hypothesis. The full procedure and results are described in the Supplementary materials (see Table S3). In addition to testing maltransfer using the between-participants manipulation, we tested for maltransfer within-participants by comparing BOTH trials to WR CONTROL trials (using the 1,200 measures of each of these trial types across 30 participants). Repeated-measures increase power (Muller et al., 1992, Guo et al. 2013).

For all analyses except reward rate, trials in which participants did not respond by the 3-second deadline were omitted (n = 36, 0.2% of all trials), as well as trials in which participants did not CN or WR, but rather pressed a key for a feature that was not displayed (n = 158, 1% of all trials). We used an alpha level of 0.05 to determine significance for all statistical tests.

#### Model-based analysis of behavior

We built on the regression analyses by fitting a Diffusion Decision Model (DDM; Ratcliff, 1978) of two-choice decision tasks to participants’ response times and percentage of goal-consistent responses (treated as a measure of accuracy). Use of the DDM allowed us to account for speed-accuracy tradeoffs and to compare directly the output of the LVOC model to the behavioral data. We fit a hierarchal DDM that simultaneously estimated parameter values for each participant, and a meta parameter for each trial type in each EITHER frequency group, using a Bayesian model fitting procedure to response times and accuracies in the Transfer Phase (HDDM version 0.6.0 in Python 3.4; Wiecki et al., 2013). The parameter of interest was the rate of evidence accumulation, or drift rate, *v*. We were particularly interested in the drift rate as an indicator for the strength of processing for either color or word relative to which response was rewarded on each trial type. We fit drift rates toward the goal-consistent response for each trial type (CN or WR, depending on which was rewarded for that trial) in each EITHER frequency group and examined group-level parameter estimates. The threshold parameter of the DDM estimates how much evidence was accumulated to reach a decision, and thus serves to index the speed-accuracy tradeoff. We fit the threshold as a function of EITHER frequency group in order to assess differences in the speed-accuracy tradeoff across EITHER frequency groups. Additional free parameters fit to each participant and the group were as follows: the trial-by-trial gaussian noise in the drift, the starting point of the drift before each trial (which can be closer to either of the two responses), and the non-decision-time that captures components of the response time not related to the decision process (such as stimulus perception and response initiation and execution). Best fit model parameters were estimated by sampling using the Markov Chain Monte Carlo (MCMC) algorithm. We used the default priors implemented in HDDM, and drew 10,000 parameter samples. The initial 5,000 samples were excluded and the remaining 5,000 samples provided a posterior distribution over parameter values on which we based our results.

We predicted that the drift rate (fit separately for each trial type in each EITHER frequency condition) would change across EITHER frequency groups. In particular, we predicted that for the BOTH trial, drift toward the goal-inconsistent response (CN) would increase with EITHER frequency (i.e., 0% group > 20% group > 50% group). We computed the difference between the posterior distribution of the 0% group and the posterior distribution of the 20% group, and the difference between the 20% group posterior distribution and the 50% group posterior distribution, for each trial type. For example, to determine whether drift rate of BOTH trials differed between the 0% group and the 20% group, we performed pairwise subtraction of the (5,000) samples of the two posterior distributions. This procedure produced a new ‘posterior difference distribution’ over the difference of 0% group minus 20% group (Kruschke, 2013). Of note we used the original order of samples for subtraction but, because MCMC samples are independent and identically distributed random variables, the result is invariant to permutations in order. We tested whether the 95% highest density interval of this posterior difference distribution contained zero. If the 95% highest density interval contained zero we concluded that there was no change from the lower (0%) to higher (20%) EITHER frequency condition. If the 95% highest density interval was strictly negative, we concluded that drift rate decreased in the higher EITHER frequency condition. Conversely, if the 95% highest density interval was strictly positive, we concluded that drift rate increased in the higher EITHER frequency conditions. In addition to testing for changes in drift rate, we tested for changes in the speed accuracy tradeoff, reflected in the threshold parameter, across EITHER frequency groups in a manner analogous to the tests of drift rate.

#### Model fit to behavior

We fit the LVOC model to the group-level drift rate estimates for each trial type in each EITHER frequency condition from our participants’ data. To do so, we maximized the likelihood of the data with respect to the model parameters using the Bayesian Adaptive Direct Search algorithm (Acerbi & Ma, 2017). The free parameters in the model were the mean and variance of the prior distribution of the feature weights, and the drift rates for the color-naming and word-reading processes. The threshold parameter and noise parameter of the LVOC model’s DDM were set to the values estimated from our participants’ data. In each simulation the threshold parameter was selected by sampling from a normal distribution reflecting the individual differences in our participants’ data. The mean and variance of the sampled distribution was set by using two parameter estimates identified by the HDDM model: the group-level estimate of mean threshold, and the group-level estimate of the standard deviation of the threshold. In each simulation the noise parameter of the DDM was sampled from a normal distribution, the mean and variance of which were set to reflect the distribution of these parameters across participants according to the HDDM analysis described above. The fitting procedure was as follows: The likelihood of the data was approximated by a product of normal distributions on the average drift rates for the different types of trials. For each potential set of parameters, the means and variances of these normal distributions were estimated by simulating the experiment 100 times; the means were computed by averaging the drift rates across all simulations; the variances of the distributions on the 30 participants’ average drift rates were computed by dividing the variance of the simulated participants’ drift rates by 30. We found that the best fitting parameters were: 1.32 as the CN drift rate (*d*_*controlled*_ = 1.32), 3.22 as the WR drift rate (*d*_*automatic*_ = 3.22), −0.17 as the mean (*μ*_*prior*_=−0.17) and 0.11 as the variance (*τ*_*prior*_=0.11) of the prior distribution of the feature weights. We then applied these best fitting parameters to a 30-participant experiment and simulated it 100 times. We performed quantitive model comparison using the Bayesian Information Criterion (BIC; Schwarz, 1978) to compare goodness of fit of the LVOC model to the behavior compared with a win-stay-lose-shift (WSLS; Restle, 1962) model that switched between CN and WR following unrewarded responses as well as a simple stimulus-response model (S-R) that learns to associate colors and words directly with responses according to a Rescorla-Wagner learning rule (Rescorla & Wagner, 1972). According to the S-R model, the association *A*_*f*, *a*_ between the feature *f* (e.g., *colorIsRed* or *wordIsBlue*) of a stimulus *s* and the participant’s response *a* is strengthened if the subsequent reward *R* is higher than expected and weakened if it is rewarded less than expected, that is

$$ {A}_{f,a}={A}_{f,a}+\alpha \cdot \left(R-\sum \limits_{f^{\prime }}{f}^{\prime }(s)\cdot {A}_{f,a}\right), $$

where *α* is the learning rate and the sum is over all features *f*^′^, which include one indicator variable for the presence of each color and one indicator variable for the presence of each word. Given the learned associations *A*_*f*, *a*_ this model then stochastically produces its response *r* (e.g., “red”) according to the exponentiated version of Luce’s choice rule, that is

$$ P\left(a|A,s\right)=\frac{\mathit{\exp}\left({\sum}_f{A}_{f,a}\cdotp f(s)\right)}{\sum_a\mathit{\exp}\left({\sum}_f{A}_{f,a}\cdotp f(s)\right)}. $$

### Summary

Our study sought to test whether people learn how to allocate cognitive control by associating stimulus features with their control-reward contingencies. To test this hypothesis, we designed a variant of the Stroop paradigm for which the LVOC model predicted maltransfer. We tasked participants with deciding whether to CN or WR on incongruent Stroop trials in two Phases and rewarded particular trials for these responses based on the color and word features of the stimuli. In the Mapping Phase, we associated a subset of colors (e.g., ) and words (e.g., **YELLOW**) with reward for CN. In the Transfer Phase, we presented novel combinations of those features and tested the LVOC models’ prediction that participants learned to CN based on those features and transferred this learning to the new stimuli (BOTH trials). If this were the case, participants should CN for the new stimuli. This would be an instance of maltransfer because WR rather than CN was the rewarded response in the Transfer Phase.

The maladaptive transfer condition was derived from the XOR rule (i.e., CN rewarded when one or the other feature is present but not both). The XOR rule cannot be learned by a strictly linear system so this condition allowed us to test both the LVOC models’ hypothesis of linearity and maltransfer. We parametrically manipulated the frequency of CN rewarded EITHER trials, which shared one feature with BOTH trials to manipulate the amount of maltransfer. Our results focus on this between-group manipulation. We specifically predicted group effects of transfer of value between trials that share features with EITHER trials (BOTH and NEITHER trials) and not for trials that did not share features with our manipulation (CN CONTROL and WR CONTROL trials). We used linear mixed-effects regression to test the effect of EITHER frequency group on responses to unrewarded features (goal-inconsistent responses). We fitted drift diffusion models to both responses and response times to test our specific hypothesis that changes in responses are due to changes in the strength of stimulus processing (drift rate; in the LVOC model cognitive control increases drift rate towards the CN response) and not other response components that could change between groups (e.g., threshold, bias, non-decision time). Furthermore, drift diffusion modeling allowed us to directly fit the LVOC model to behavior since responses in the LVOC model were simulated using a drift diffusion process.

## Results

**Figure 2.**
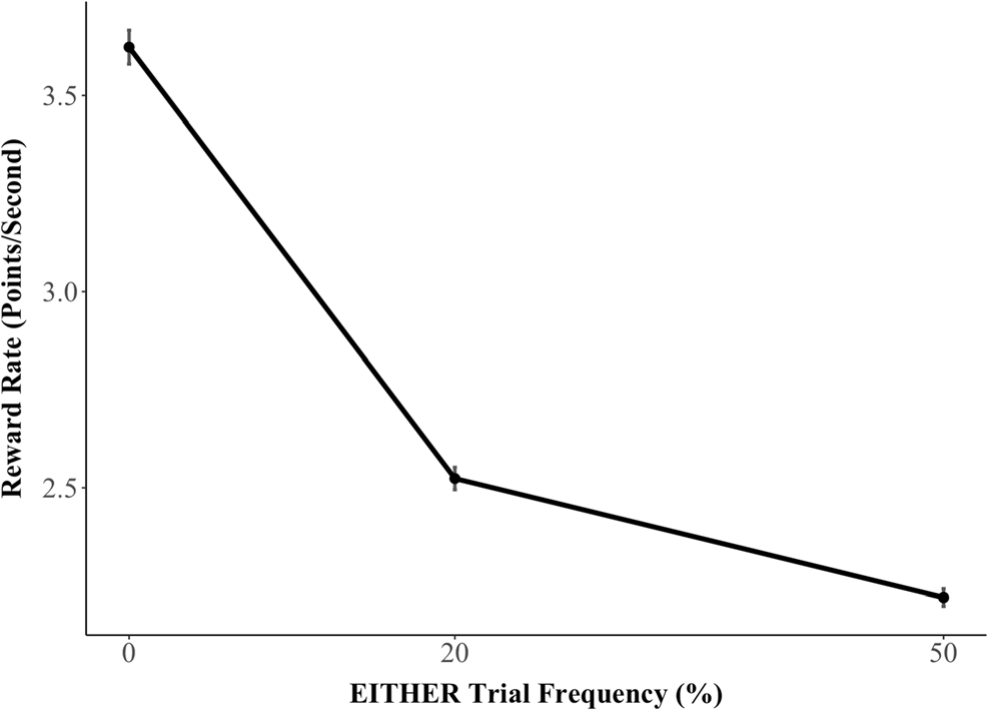
Reward rate in the Transfer Phase by experimental group (i.e., frequency of EITHER trials) in human behavior. Consistent with LVOC model prediction, Transfer Phase reward rate decreased with an increase in EITHER trial frequency. Error bars indicate standard errors of the mean.

The LVOC model predicted that transfer effects in our experiment would result in an overall decrease of reward rate in the Transfer Phase for higher EITHER frequency groups. Regression modeling confirmed this prediction, showing a significant deleterious effect of the proportion of EITHER trials on the frequency of goal-inconsistent responses (β = 0.03, *SE* = 0.00, *Z* = 8.28, *p* < 0.001) and participants’ reward rate (β = −0.03, *SE* = 0.00, *t* = −7.30, *p* < 0.001; Figure 2). According to the LVOC model, this decrement in performance was due to maltransfer to trial types that shared features with EITHER trials (rewarded for CN) but for which WR was the rewarded response (BOTH and NEITHER trials). We tested this account by examining each trial type individually.

**Figure 3.**
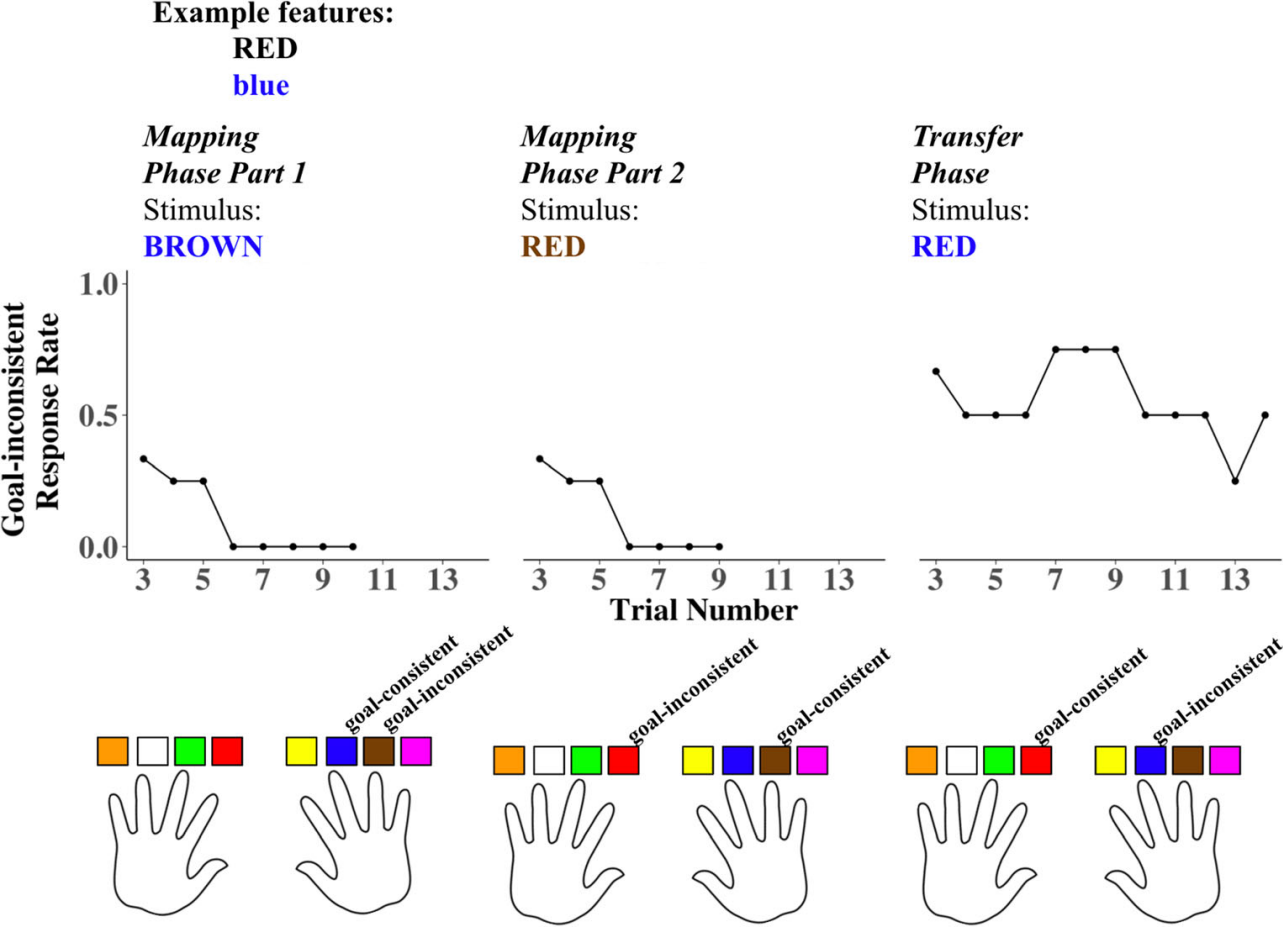
Responses of a single participant (9) to example set of features throughout the experiment. Data plots show a three trial moving average of goal-inconsistent response rate for three stimuli containing the features **RED**, and . Bottom graphic indicates which button press response was rewarded (goal-consistent) for each stimulus.

Because the LVOC model learns to approximate the value of exerting control as a linear combination of stimulus features, it predicts maltransfer between stimuli that share a feature (i.e., their color or word) but that differ in their demand for controlled versus automatic processing. For BOTH trials, each of the individual stimulus features was mapped to CN during the Mapping Phase (e.g., font color  and word **RED**), but their combination was mapped to WR in the Transfer Phase (e.g., ). The key prediction for the present experiment is that performance on BOTH trials would be impaired by learned mappings acquired in both parts of the Mapping Phase and that this impairment increases with the frequency of EITHER trials (e.g., ), which shared one feature with BOTH trials (e.g., ). This prediction is exemplified by the responses of participant 9 (from the 50% EITHER trial frequency group) to a selected set of features over the course of the experiment (Figure 3).

**Figure 4.**
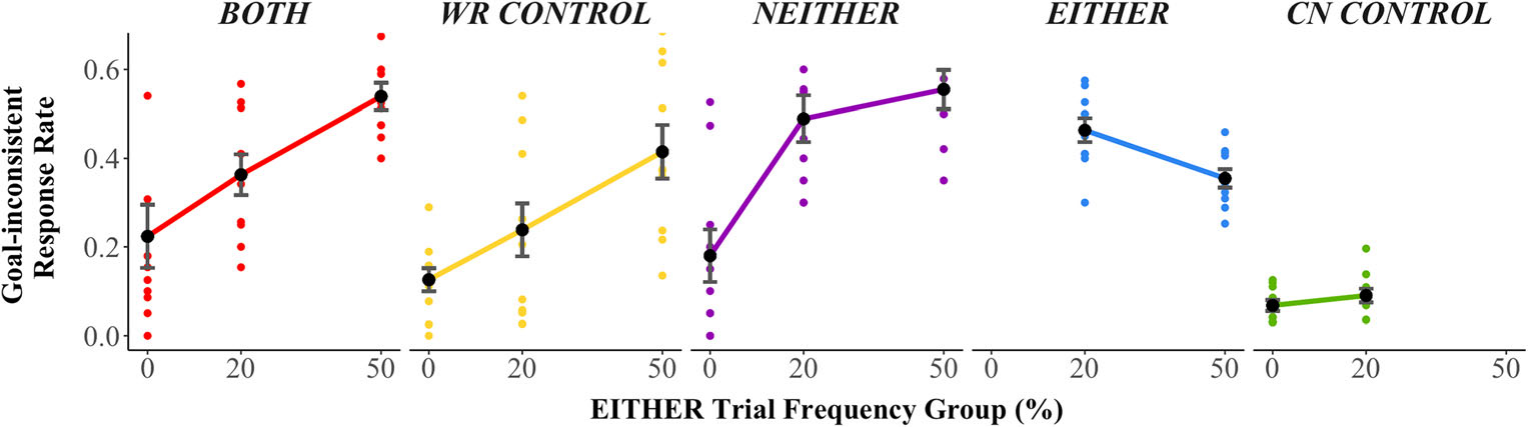
Goal-inconsistent response rate by experimental group and trial type. Goal-inconsistent response rates for BOTH (red), NEITHER (purple), and WR CONTROL trials (yellow) were greater when EITHER trials were more frequent. Colored points are the mean goal-inconsistent response rate per participant. Black points are the mean across participants. Error bars indicate standard errors of the mean.

This occurs in the model because (i) the weights of the stimulus features present in BOTH trials increase during exposure to EITHER trials in the Mapping Phase, and (ii) the weights of features shared between EITHER trials and BOTH trials increase with the frequency of EITHER trials. Consistent with the LVOC model’s predictions, there was a significant increase in goal-inconsistent responses (CN responses) on BOTH trials with increasing frequency of EITHER trials (β = 0.03, *SE* = 0.01, *Z* = 4.38, *p* < 0.001; Figure 4).

**Figure 5.**
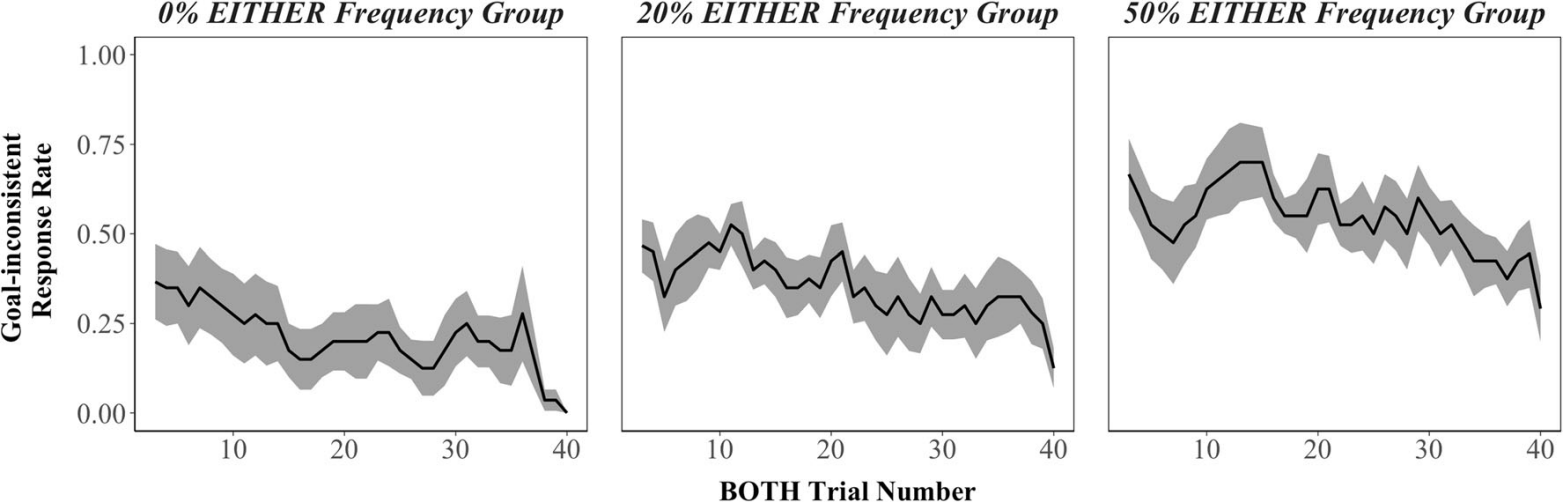
BOTH trial goal-inconsistent response rate across Transfer Phase. Three trial moving average of goal-inconsistent response rates for BOTH trials. Goal inconsistent responses remained elevated throughout the Transfer Phase. Black lines indicate mean goal-inconsistent response rates across participants. Error bars indicate standard errors of the mean.

Despite 40 trials of experience with the BOTH condition, the effect of maltransfer persisted throughout the Transfer Phase (Figure 5). Participants in all groups improved on BOTH trials over the course of the Transfer Phase (fewer goal-inconsistent responses, β = −0.035, *SE* = 0.0098, *Z* = −3.55, *p* < 0.001). This was likely due to gradual learning of a combined feature representation (e.g., *f*(*s*, *c*) = *colorIsBlue*(*s*, *c*) · *wordIsRed*(*s*, *c*)) that allowed them to learn the reward contingencies for BOTH trials with reduced maltransfer interference.

To test whether the BOTH maltransfer effect was feature-specific, we compared goal-inconsistent response rates of BOTH trials to WR CONTROL trials. As the LVOC predicted, performance on BOTH trials was worse than WR CONTROL trials (more goal-inconsistent responses, β = 0.66, *SE* = 0.21, *Z* = 5.52, *p* < 0.002). This within-participants contrast provides evidence of maltransfer, complementing the between-participants EITHER frequency group effect.

**Figure 6.**
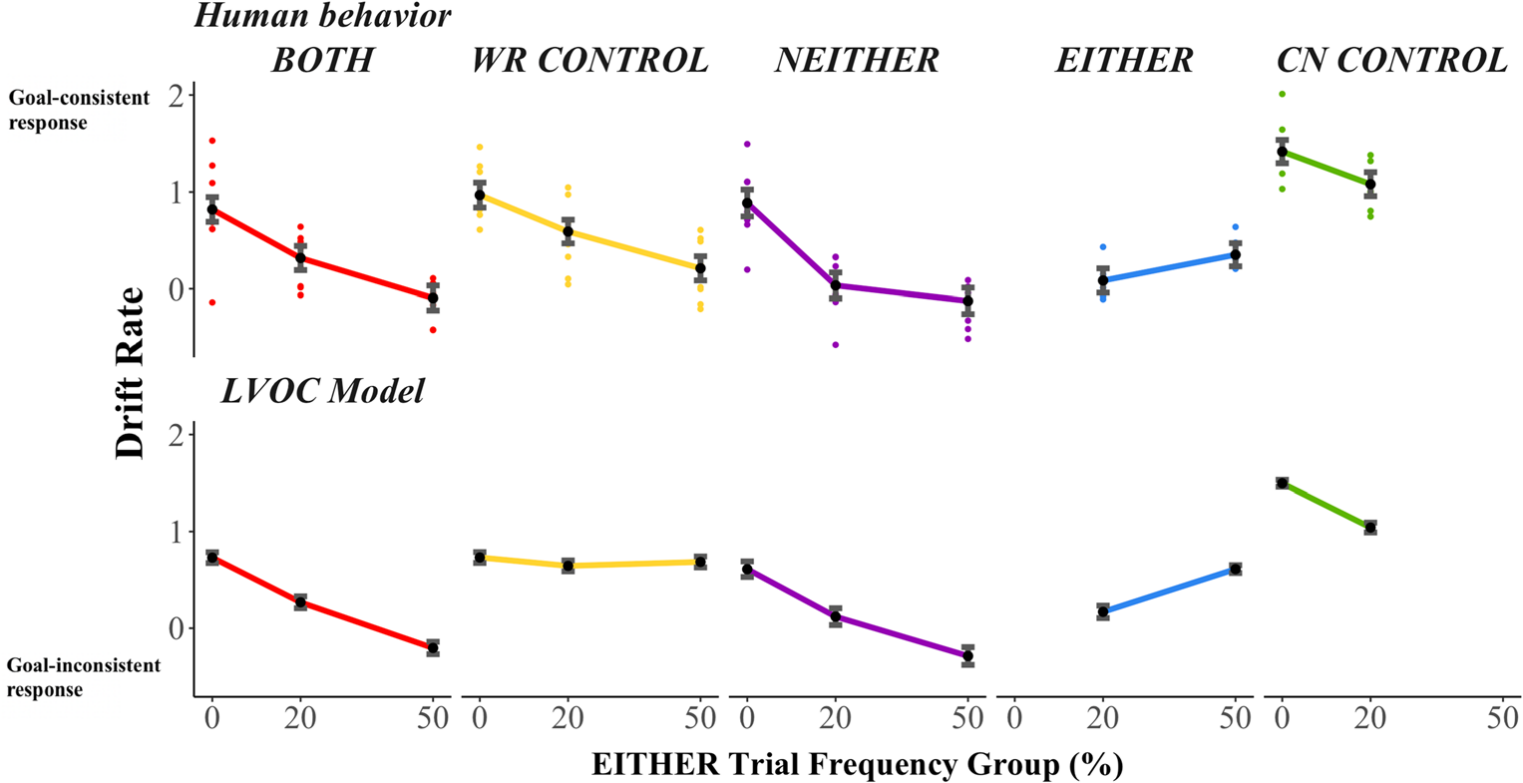
Drift rates fit directly to behavior and determined from LVOC model fit to behavior. Top panel: drift rates estimated from HDDM fit to behavior by trial type and EITHER frequency group. Black points are mean of samples of the group-level parameters (error bars indicate standard deviation of group-level parameter). Colored points are the mean of samples of the participant-level parameters. Drift rates are toward the goal-consistent (rewarded) response. For example, for BOTH trials, drift rate towards the WR response was smaller when EITHER trials were more frequent. For EITHER trials, drift rate toward the color-naming response was greater when EITHER trials were more frequent. Bottom panel: drift rate from LVOC model applied across Transfer Phase (error bars indicate standard error of the mean across simulations). LVOC model captures qualitative effects in group-level drift rates observed in the behavioral data for all trial types except WR CONTROL trials (yellow).

The HDDM fit to behavior suggested that increased strength of processing of the color feature was responsible for the increased propensity to CN for BOTH trials in higher EITHER frequency groups (for exact values of goal-inconsistent response rates and HDDM parameters, see Table S1). The HDDM model samples converged for all parameters, and simulations from the fitted model closely matched participant goal-inconsistent response rate, and mostly captured response time patterns (Figures S2 and S3). The HDDM fit indicated that drift rate towards the goal-consistent response (WR) decreased and nearly reversed toward the goal-inconsistent response in higher EITHER frequency groups, and the LVOC model fit to the HDDM parameters captured this trend (Figure 6). Consistent with the observation that the mean value of the posterior distribution of drift rates was lower in higher EITHER frequency groups, we found that the 95% highest density interval of the posterior difference distribution over the drift rate for BOTH trials in the 0% group minus the 20% group was strictly negative and did not contain zero (Table 4). The same decrease was found for the posterior difference distribution over the drift rate of BOTH trials in the 20% group minus the 50% group (Table 4).

**Table 4.** Test for change in HDDM parameters across EITHER frequency groups. Rows; HDDM parameter examined. Second column; 95% highest density interval for posterior difference distribution over MCMC samples of parameter in 0% minus 20% EITHER frequency group. Third column; 95% highest density interval for posterior difference distribution over MCMC samples of parameter in 20% minus 50% EITHER frequency group. Interval containing zero suggests no change in the parameter across groups. Negative values suggest that the parameter is lower in the higher compared with the lower EITHER frequency group.

HDDM parameter	Frequency of EITHER trials	
	Difference between 0% and 20% groups	Difference between 20% and 50% groups
Threshold	−0.20 to 0.25	−0.49 to −0.04
BOTH drift rate	−0.86 to −0.15	−0.76 to −0.05
NEITHER drift rate	−1.23 to −0.48	−0.55 to 0.22
EITHER drift rate	—	−0.07 to 0.61
WR CONTROL drift rate	−0.73 to −0.03	−0.72 to −0.04
CN CONTROL drift rate	−0.67 to −0.01	—

Performance in BOTH trials, both the within- and between-participants, is consistent with predictions made by the hypothesis that feature-based maltransfer results from learned values of control (CN). The LVOC model also makes specific predictions for each of the other trial types in this experiment (Figure 6). Across all of these trial types, we found evidence that all stimuli that shared a feature with EITHER trials were subject to transfer learning. In addition, we found evidence for a representation of the general value of control over the experiment as a whole. Bayesian analysis showed that the number of data points we collected was sufficient to obtain substantial, strong, or decisive evidence for or against every each of the effects we considered, according to the standard interpretation of Bayes factors introduced by Kass and Raftery (1995) (Table S3). We consider each of the trial types individually and in greater detail below.

First, NEITHER trials were trials in which neither of stimulus features were mapped to CN in the Mapping Phase (e.g., ) nor in the Transfer Phase (e.g., ), but rather to WR. Therefore, Mapping Phase training should have benefitted Transfer Phase performance. However, NEITHER trials (e.g., ) also shared the WR-mapped feature of EITHER trials (e.g., ), for which the rewarded task is CN. The LVOC model therefore predicted maltransfer (increased CN) from EITHER trials to NEITHER trials. In line with the LVOC model’s predictions, participants who were exposed to more EITHER trials were more likely to CN on NEITHER trials and consequently gave more goal-inconsistent responses (Figure 4; β = 0.04, *SE* = 0.01, *Z* = 4.31, *p* < 0.001). For NEITHER trials, the HDDM results indicated that drift rate towards the goal-consistent response decreased in the 20% compared with 0% EITHER frequency condition, but not in 50% EITHER frequency condition compared with the 20% EITHER frequency condition (Figure 6; Table 4). We predicted that NEITHER trials would show less maltransfer than BOTH trials, because there was no interference from Mapping Phase training to NEITHER trials (each of the features were WR-mapped and remained so in the Transfer Phase). Comparing NEITHER trials to BOTH trials (for which Mapping Phase was predicted to be detrimental), we found no evidence for a difference in goal-inconsistent response rate (BOTH trials did not have a significantly different rate of goal-inconsistent responses, β = −0.16, *SE* = 0.15, *Z* = 4.94, *p* < 0.273). It is worth noting that NEITHER trials were the least frequent trial type, which may have diminished potential benefit from the Mapping Phase transfer (Figure 4). The LVOC model fit to the HDDM results was able to capture the drift rate effects for NEITHER trials (Figure 6).

The increased frequency of EITHER stimuli across groups was balanced by a decreased frequency of CN CONTROL trials to maintain a constant overall balance of CN rewarded versus WR rewarded trials. EITHER trials (e.g., ) did not share features with CN CONTROL trials (e.g., ). The LVOC model would predict that, for both of these trial types the proportion of goal-consistent responses should be higher when that trial type is more frequent. Evidence was mixed as to whether performance on EITHER trials improved as they became more frequent. Regression analyses confirmed that goal-consistent (CN) responses for EITHER trials increased in higher EITHER frequency groups (Figure 4: fewer goal-inconsistent responses; β = −0.02, *SE* = 0.00, *Z* = −3.22, *p* = 0.001); however, HDDM model-based results did not indicate a statistically significant change in drift rate across groups. Although the average of the posterior distribution indicated that drift rate toward the rewarded response increased (Figure 6), the posterior difference distribution contained zero (Table 4). Evidence also was mixed as to whether CN CONTROL trial performance decreased with increasing frequency of EITHER trials due to less experience. Regression analysis indicated no reliable change in goal-inconsistent response rate for CN CONTROL trials across EITHER frequency groups (β = 0.016, *SE* = 0.01, *Z* = 1.22, *p* = 0.222). The LVOC model fit captured the drift rate patterns for both EITHER and CN CONTROL trial types (Figure 6). However, HDDM model-based results for CN CONTROL trials revealed that drift rate toward the goal-consistent (CN) response decreased in higher EITHER frequency groups (for both 0% frequency compared to 20% frequency, and 20% frequency compared to 50% frequency, 95% highest density interval of the posterior difference distribution was negative and did not contain zero; Table 4).

The only trial type for which human performance was not captured by the LVOC model was WR CONTROL trials. WR CONTROL trials (e.g., ) did not share features with EITHER trials (e.g., ), and WR was the rewarded response. Therefore, the model did not predict maltransfer from EITHER trials to WR CONTROL trials based on stimulus features alone. This is because they did not share any stimulus-specific features. Contrary to this prediction, goal-inconsistent (CN) responses on WR CONTROL trials increased as the frequency of EITHER trials increased (Figure 4: β = 0.03, *SE* = 0.01, *Z* = 4.08, *p* < 0.001). HDDM results corroborated this finding; drift rate decreased toward the goal-consistent response in higher EITHER frequency groups (Figure 6). This decrease was reflected in the WR CONTROL drift rate posterior difference density over 0% group minus 20% group, which was strictly negative, as was the posterior difference density over 20% group minus 50% group (Table 4). According to the LVOC model, the only feature that connects WR CONTROL and EITHER trial types is that of the global value of control for the situation. This result is consistent with that account that the overall learned value of control (i.e., CN) was greater in higher EITHER frequency conditions (despite the actual reward available for control being balanced). This might arise if people learn a general value for control more readily than a value for control contingent on stimulus-specific features. This account is consistent with the idea that, with sparse data, learning in neural systems is biased toward more general (i.e., shared) characteristics of a task situation, and only with more training do they develop dedicated (i.e., distinct or separated) representations for more specific features of the circumstance (Caruana; 1997; Musslick et al.; 2017; Saxe, McClelland, & Ganguli, 2019). We analyze a modification of the LVOC model to account for this discrepancy after we compare the original LVOC model to two alternatives in the Model fit and model comparison section below.

### Model fit and model comparison

We found that the LVOC model was able to fit accurately the drift rates of each trial type except the WR CONTROL trials (Figure 6). To determine that the complexity of the model was justified by its fit to the data, we performed quantitative model comparisons against an alternative Win-Stay Lose-Shift (WSLS) model that switched between CN and WR following unrewarded responses and repeated its choice following rewarded responses. Our analyses indicated that the LVOC model explained the data significantly better than the simpler alternative model (*BIC*_*LVOC*_ = 4.5 vs. *BIC*_*WSLS*_ = 707.8). Furthermore, the LVOC model explained the goal-inconsistent response rates observed in our experiment significantly better than the simple Stimulus-Response (SR) model according to which people would learn to directly associate colors and words with button presses (*BIC*_*LVOC*_ = 1064.2 vs. *BIC*_*SR*_ = 3309.2).^1^

**Figure 7.**
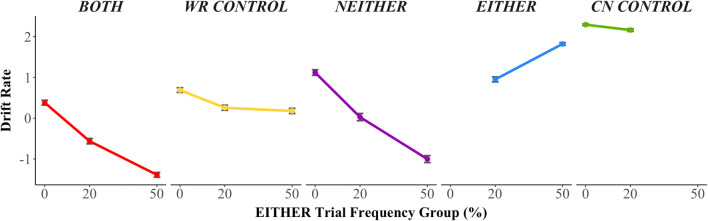
Results of LVOC model with faster learning of global value of control. Drift rate for WR CONTROL trials (yellow) decreases with increasing EITHER frequency when the LVOC model is biased toward learning a general value of control more quickly than feature-specific values.

As discussed earlier, for WR CONTROL trials, the LVOC model did not capture the decrease in drift rate with increased frequency of EITHER trials. We speculated that this discrepancy may be due to a difference in the way the global weight for the value of control was being learned relative to weights for stimulus features. To test this in the LVOC model, we allowed the global weight to be learned more rapidly than other weights. We did so by increasing the prior precision of the global weight,^2^ which allows faster learning, because there is less uncertainty. Importantly, this mechanism is agnostic to the direction in which the global weight should change. We found that this manipulation allowed the model to capture the pattern of performance in the WR CONTROL trials, as well as all of the other effects previously exhibited (*BIC*_*LVOC global bias*_ = 11.43; Figure 7).

## Discussion

The experiment reported here was designed to test predictions of the LVOC model of how people learn to allocate control. This model assumes that they do so by learning associations between stimulus features and the expected value of control (EVC), based on the reward received from allocating control in response to particular stimuli. Thus, the model predicts that stimulus features that are consistently associated with reward for allocating control (in excess of the costs of doing so) should generate a high EVC whenever those features appear in a stimulus and thus favor the allocation of control. Critically, because the LVOC is a strictly linear model, it also predicts that if two different stimulus features both are associated with a high EVC, favoring the allocation of control, then their simultaneous presence in the stimulus also should (even more strongly) favor the allocation of control (assuming they have not appeared together before). To test these predictions, we used a variant of the Stroop paradigm in which specific individual features (colors and words) were associated with reward for either CN or WR. Participants were trained on these individually and then tested on combinations of these features. In addition, we explicitly manipulated the frequency with which particular features were associated with reward for CN across three groups of participants, as a parametric test of the effects of learning. Our analyses of the data included: i) a regression analyses of response time and accuracy responding to the rewarded dimension; ii) a fit of the drift diffusion model (using the HDDM) to those data; iii) use of the parameter estimates from the fit of the HDDM to compare human performance with quantitative predictions of the LVOC model generated in simulations of the experimental task; iv) and formal model comparison of the LVOC to two simpler models with respect to their ability to fit the empirical data.

As predicted by the LVOC model, we found that human participants showed a significant propensity to choose the control-demanding response (CN) for stimuli (BOTH trials) combining features that were previously associated with higher reward for the controlled (CN) response (in the Mapping Phase), even when there was greater reward for selecting the less demanding (WR) response for that combination of features (in the Transfer Phase)—that is, maltransfer. The finding of maltransfer is particularly striking, because people generally avoid exerting cognitive control (Kool et al. 2010, 2013; Westbrook et al. 2013). Our findings strongly suggest that people learn how to allocate cognitive control and generalize what they have learned to novel situations. At the same time, the behavioral results suggest that people use relatively simple learning rules (such as the one implemented in the LVOC, which is linearly additive) that, at least in this experiment, did not show sensitivity to nonlinear associations (i.e., the XOR rule used to determine the rewarded response in the Transfer Phase). In addition to the present experiment, the LVOC model has been successfully applied to five other experiments in the literature measuring adaptive, experience-driven changes in how people allocate cognitive control (Lieder et al., 2018). Each of the five experiments exposed situations in which participants had to learn about the value of control. The five experiments differed in which stimulus features were associated with a high value of control (e.g., the color of a Stroop stimulus or the semantic category of a picture) and the types of tasks that required control allocation (color naming or categorizing animals). In all of these previous experiments, learning to predict the value of control from a linear combination of features improved task performance, or reward rate. The present experiment complements these findings by demonstrating that, by exploiting the limitations of the learning mechanism, the effects of learning can be observed even when they are maladaptive.

Importantly, the maltransfer predicted by the LVOC model is a result of its simplicity, which was to a large extent observed in the empirical data. This suggests that the LVOC model can be used to uncover sources of real world cognitive control failures. For example, the linearity of the learning rule implemented in the LVOC model predicts maltransfer where there are nonlinearities in the value of control, such as the ones tested in our experiment. Such nonlinearities may arise in other settings, such as in multitasking, where the value of allocating cognitive control to each of two activities on its own (e.g., driving or texting) does not accurately predict the value of allocating control to both simultaneously (e.g., texting *and* driving). Thus, the LVOC model may be useful for understanding maladaptions of control in these and other settings. Understanding biases in cognitive control plasticity could be leveraged to design interventions that help people learn how to pursue their goals more effectively by adapting what they attend to, and how they make their decisions.

Based on previous work (Shenhav et al., 2013, 2017), we suggest that the approximate cost-benefit analysis assumed by the LVOC model is performed by the dorsal Anterior Cingulate cortex, and that the control allocation itself is implemented by the dorsolateral prefrontal cortex (Miller & Cohen, 2001; Badre, 2008). These predictions could be explored by regressing variables and parameters of the LVOC model against neural dynamics (such as BOLD) in these two brain areas that unfold over the course of learning how to allocate control.

Our experiment also included trials that probed global transfer of the value of control. Surprisingly, these trials also yielded evidence for the overallocation of control. Post-hoc modeling was consistent with the suggestion that overgeneralization of the value of control may reflect a bias toward learning lower dimensional, general-purpose representations of the value of control (i.e., the feature-independent value of control), over learning higher dimensional, more specific representations (i.e., the predicted value of control for individual features, such as the word **RED**, and the value for of specific combinations of features, such as the stimulus ). Although this was not a prediction we made *a priori*, it is consistent with theoretical accounts of learning in other domains, such as learning of semantic categories or in multitask learning. In category learning, an agent is presented with specific exemplars and is tasked with grouping the exemplars into categories. Accounts of how this learning is accomplished suggest that there is a bias toward learning the simplest categories that can explain exemplars before being driven to learn more complex, feature-specific categories (Feldman, 2003; Goodman et al., 2008; Rogers & McClelland, 2004; Saxe, McClelland, & Ganguli, 2013, 2019). The learning of low-dimensional representations also are the target of various machine learning techniques, including multitask learning. In the multitask learning paradigm, a learner is trained to perform multiple tasks with distinct input-output relationships. Researchers have found that when the network uses an overlapping set of units and weights to accomplish multiple tasks (“shared representations”), as opposed to using task-dedicated, nonoverlapping units (“separated representations”), the network will learn more quickly and will better generalize what it has learned when it encounters a new task (Caruana, 1997; Baxter, 1995; Musslick et al., 2017). These principles of faster learning and improved generalization also may apply to the case of learning about the value of control, suggesting that people may initially be more sensitive to and more quickly learn general characteristics of the control-requirements of a situation, before learning subtler, more specific characteristics.

The LVOC model is similar to a number of other models that adaptively adjust cognitive control allocation, including the conflict adaptation model (Botvinick et al., 2001), a Hebbian Learning account of conflict adaptation (Verguts & Notebaert, 2008), and others (for a review see Jiang, Heller, & Egner, 2014). Nevertheless, there are differences. In the conflict monitoring model, changes in control allocation across trials are proportional to the amount of response conflict that the model registers on the previous trial. In the LVOC model, adaptations of control across trials are the result of trial-by-trial learning of the LVOC (through Bayesian regression). The mechanism underlying control adaptation in the conflict monitoring model might be regarded as an efficient approximation of the feature-based learning mechanism of the LVOC model. However, unlike the LVOC model, the conflict monitoring model fails to capture feature-specific adaptations to response conflict. Verguts & Notebaert (2008) proposed that such feature-specific adaptations may be achieved through Hebbian learning. Similarly, Jiang and colleagues (2014) proposed a Bayesian model of context-based learning about control demands of the environment. Their “Bayesian model of flexible cognitive control” learns about demands for control for short- and long-time scales, as well as a belief about the volatility of control demands. It uses its belief about the volatility to weight short- and long-time scale information to predict and deploy control. The model has empirical support in experiments manipulating the proportion of incongruent stimuli over time and captures classic sequence congruency effects (Jiang et al., 2014; Jiang, Brashier, & Egner, 2015; Muhle-Karbe et al., 2018; Jiang et al., 2020). This model and empirical results provide additional support for the hypothesis that people learn to exert control using contextual features including a global value of control. The benefit of the model by Jiang et al. (2014) is that it can capture the effect of the volatility in control demands of the environment. In comparison, the benefits of the LVOC model include: i) it can capture how people combine multiple features of the stimulus presented on the current trial to anticipate the value of control; ii) it can capture feature-specific transfer effects; iii) it can capture the effect of different levels of reward magnitude on learning and control adjustment; and iv) it is simple and may explain biases in learning.

One limitation of the proposed model is the selection of relevant features by the modeler. Moreover, the LVOC model described here may not be able to learn complex nonlinear relationships between features and the value of control, even when given substantial experience with these. The learning of such relationships can be accomplished in more complex, multilayer nonlinear neural networks. The input layer of such a network may encode raw features of the environment (such as the pixels of the experiment screen). These input features may then project through one or more intermediate (hidden) layers to a single output unit representing the value of control. Analogous to Bayesian Linear Regression, the network could be trained to predict the value of control in such situations through supervised learning (e.g., backpropagation; Rumelhart et al., 1986) from raw stimulus features. Unlike linear regression, the network would be able to discover and represent task-relevant features that are not hand-coded into the network and that would allow the network to better predict the value of control. Nevertheless, as suggested above, like our modified version the LVOC model, it may still show an initial bias to learn simpler, more general features of a circumstance that demand control before learning more complex, nonlinear relationships. An extension of the LVOC to such models may be able to capture nonlinear and arbitrary interactions among the features at various levels of generalization while exhibiting the full pattern of empirical results we observed, without the need for the post-hoc modification to the LVOC to capture the data. However, unlike the LVOC model presented here, such an extension would lack interpretability due to nonlinearities that operate on a high number of parameters.

An experimental extension of this work could test whether the LVOC predictions hold in a setting where rules are not as heavily, or even ever, based on single features. Reward contingencies in the Mapping Phase of the present experiment were based on single features, which may have biased participants towards single feature learning, whereas in the Transfer Phase reward contingencies were based on combinations of features. Future experiments could manipulate the degree to which reward contingencies are based on single features versus combinations of features (for example, including only the Transfer Phase trial types from this study) to determine parametrically the extent to which people are biased toward the learning of rules based on single features versus combinations of them. The present study had 30 participants. Bayes factor analysis showed that the data from this study provided substantial, strong, or decisive evidence for or against each of the null-hypotheses that we considered. Based on our findings, the present experiment merits replication in a larger sample size, which also could be used to explore individual differences.

The mechanism postulated by the LVOC model could coexist with other mechanisms for selecting control signals. The LVOC mechanism can be considered a “model-free” reinforcement learning mechanism that might be complemented by a mechanism that computes the EVC based on a more sophisticated, model-based evaluation of the effects of alternative allocations of cognitive control (Musslick et al., 2015). One challenge for future development of any of these models of EVC computation is scalability. For the LVOC model, the number of weights that have to be learned increases factorially with the number of stimulus dimensions and control signals that must be attended (e.g., in the present experiment, control demands could have been based not just on word and color features, but also on font, font size, and font weight). It is likely that attention serves to constrain the space of features used for learning (Gershman & Niv, 2010); however, this begs the question of what determines such attentional focus. In general, much remains to be investigated about meta-learning problems, such as which features to select or attend and how optimally to prioritize learning of more general versus more specific features.

## Supplementary Information

ESM 1 (DOCX 1596 kb)

## Footnotes

1 LVOC model was fit to different data for the WSLS and SR model comparisons. For WSLS vs. LVOC, the models were fit to drift rates. For SR vs. LVOC, the models were fit to trial specific stimuli and goal-consistent/inconsistent responses.

2 Specifically, we set the precision of the model’s prior on the weights of feature and feature conjunctions (64.9) to be significantly less precise than the prior on the global weight for control signal intensity (21.1336). The mean of the model's prior on all weights favored the automatic process (−0.17), and the drift rates were unchanged from the original model fit.
