# Supplementary material for: Learning to Overexert Cognitive Control in a Stroop Task
Source: Cogn Affect Behav Neurosci. 2021 Jan 6;21(3):453–71. doi: 10.3758/s13415-020-00845-x (PMC8208940; doi:10.3758/s13415-020-00845-x)
Supplement: Supplementary file 1 — (DOCX 1596 kb) [file 13415_2020_845_MOESM1_ESM.docx]

**Supplementary**

**Additional task information**

The experiment was implemented using Matlab (version 2016a) with the toolbox Psychtoolbox (version 3.0.13, <http://github.com/Psychtoolbox-3/Psychtoolbox-3>). The main task took about 20 minutes. Each Mapping Phase took about 6 minutes with a self-paced break in-between, and the Transfer took about 8 minutes with a break halfway through.

|  | **Frequency of EITHER trials** | | | | | | | | | |
| --- | --- | --- | --- | --- | --- | --- | --- | --- | --- | --- |
| A. Each trial type | 0% EITHER | | | 20% EITHER | | | | 50% EITHER | | |
|  | **0%** | | | **20%** | | | | **50%** | | |
|  | goal-inconsistent response rate (%) | response time | drift rate | goal-inconsistent response rate (%) | | response time | drift rate | goal-inconsistent response rate (%) | response time | drift rate |
| BOTH | 22.37 ± 22.61 (*SEM*) | 1270 ± 246 (*SEM*) | 0.82 ± 0.13 (posterior *SD*) | 36.30 ± 14.55 | | 1509 ± 234 | 0.32 ± 0.13 | 53.92 ± 9.72 | 1307 ± 282 | -0.09 ± 0.13 |
| NEITHER | 18.00 ± 18.78 | 1272 ± 245 | 0.89 ± 0.14 | 48.92 ± 16.65 | | 1520 + 265 | 0.03 ± 0.13 | 55.50 ± 13.93 | 1335 ± 265 | -0.12 ± 0.13 |
| EITHER | - | | | 46.36 ± 8.55 | | 1535 ± 257 | 0.09 ± 0.13 | 35.48 ± 6.59 | 1303 ± 227 | 0.35 ± 0.12 |
| WR CONTROL | 12.57 ± 8.25 | 1260 ± 165 | 0.96 ± 0.13 | 23.85 ± 18.92 | | 1548 ± 236 | 0.59 ± 0.12 | 41.49 ± 19.16 | 1392 v 259 | 0.21 ± 0.13 |
| CN CONTROL | 6.75 ± 3.90 | 1044 ± 110 | 1.42 ± 0.12 | 8.99 ± 4.85 | | 1374 ± 244 | 1.08 + 0.12 | - | | |
| **B. All trial types** | **0% EITHER** | | | **20% EITHER** | | | | **50% EITHER** | | |
| Overall goal-inconsistent response rate | 12.12 ± 7.52 (*SEM*) | | | 29.04 ± 5.88 | | | | 42.43 ± 5.51 | | |
| Reward rate (points/second) | 3.33 ± 0.04 (*SEM*) | | | 2.35 ± 0.03 | | | | 2.07 ± 0.02 | | |
| DDM Threshold | 2.27 (*M*) | | | 2.30 | | | | 2.05 | | |
| DDM Threshold SD | 0.24 | | | 0.24 | | | | 0.24 | | |
| **C. All EITHER frequency groups** | | | | | | | | | | |
| DDM non-decision time (*M*) | | | | | 0.40 | | | | | |
| DDM standard deviation of individual participant non-decision time parameter around meta parameter | | | | | 0.29 | | | | | |
| DDM drift noise (*M*) | | | | | 0.24 | | | | | |
| DDM standard deviation of individual participant drift rate parameter around meta parameter | | | | | 0.34 | | | | | |

**Supplementary Table 1:** *Summary of regression and DDM analyses.* Panel A: By trial type (rows), by EITHER frequency condition (columns), summary statistics of the percent of goal-inconsistent responses and response times, and DDM model-based average drift rate. Panel B: Grouped by EITHER frequency condition (columns), summary statistics of overall goal-inconsistent response rate and overall Transfer Phase reward rate, and DDM model-based measure of threshold and standard deviation of individual participants threshold parameter around meta parameter. Panel C: For all EITHER frequency conditions, DDM-model average of posterior for non-decision time parameter, and drift noise parameter, as well as parameter for standard deviation of individual participants non-decision time parameter and drift noise parameter.

**Response time analyses**

We used mixed effects of regression to analyze the effect of EITHER frequency group on log transformed response time for each of our Transfer Phase trial types. EITHER frequency group was treated as a continuous variable and subjects were taken as random effects where each subjects’ error estimates varied around a group mean estimate. For BOTH trials we found no reliable change in RT by EITHER frequency group (β = -0.000, *SE* = 0.002, *t*(28) = -0.068, *p* = 0.95). Neither did we find a change in RT for NEITHER trials (β = 0.000, *SE* = 0.001, *t*(28) = 0.088, *p* = 0.931) nor WR CONTROL trials (β = 0.002, *SE* = 0.002, *t*(28) = 1.181, *p* = 0.248). Response time on EITHER trials decreased across EITHER frequency groups (β = -0.01, *SE* = 0.00, *t*(18.10) = -2.13, *p* < 0.048). Response time on CN CONTROL trials increased across EITHER frequency groups (response time increased; β = 0.014, *SE* = 0.003, *t*(17.68) = 4.14, *p* < 0.001). In addition we separated trials in which participants made the goal-consistent response and the goal-inconsistent response (Figure S1) and found the same qualitative pattern of results.


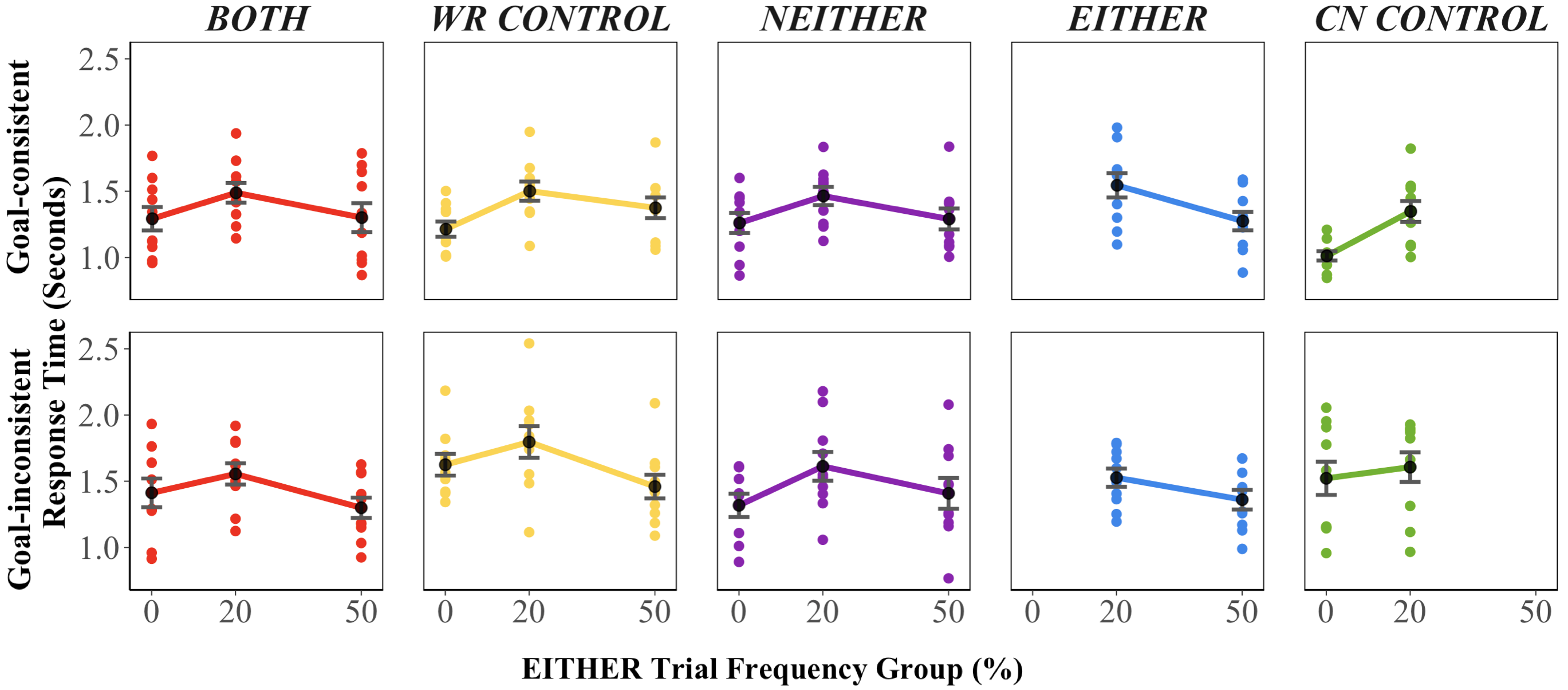


**Supplementary Figure 1:** *Behavior response time by trial type and EITHER frequency group.* Top, goal-consistent trials. Bottom, goal-inconsistent trials. Colored points indicate the mean response time per participant. Black points indicate the mean across participants. Error bars indicate standard errors of the mean.

**Additional Drift Diffusion Model analyses**

In the Results section we reported the average value for the HDDM fitted drift rate (Table 4). This number was derived from a bayesian estimated posterior density of parameter values from 5,000 MCMC samples. The range of these values indicates our uncertainty in parameter values. We computed the 95% highest density intervals which indicate that we are 95% confident that the true parameter value is in this range (values in Table S2).

| **HDDM parameter** | **Frequency of EITHER trials** | | |
| --- | --- | --- | --- |
|  | **0%** | **20%** | **50%** |
| threshold | 2.11 to 2.44 | 2.14 to 2.47 | 1.89 to 2.21 |
| BOTH drift rate | 0.57 to 1.07 | 0.07 to 0.56 | -0.34 to 0.16 |
| NEITHER drift rate | 0.61 to 1.16 | -0.23 to 0.29 | -0.39 to 0.13 |
| EITHER drift rate | - | -0.16 to 0.33 | 0.11 to 0.59 |
| WR CONTROL drift rate | 0.72 to 1.22 | 0.34 to 0.83 | -0.03 to 0.46 |
| CN CONTROL drift rate | 1.18 to 1.65 | 0.84 to 1.32 | - |

**Supplementary Table 2**: *95% highest density interval for HDDM parameters*.

We also assessed the HDDM fit to the behavior data by simulating data from the fitted model. Using the HDDM method ‘Posterior Predictive Checks’ we simulated responses and response times from each participant and each trial type (2,897,500 total, approximately 97,000 per participant). Then we aggregated the data to compute goal-inconsistent response rates (Figure S2) and response times (Figure S3) to compare to the human behavior data. The simulation closely corresponded to the goal-inconsistent response rate of behavior data. The simulation captured many patterns of the response time results, but produced similar patterns for goal-consistent and goal-inconsistent trials, whereas participants were slower on goal-inconsistent versus goal-consistent trials. The behavioral phenomenon of slow errors is well known, and the classical DDM cannot capture this asymmetry (Fard et al. 2017). To capture that effect would require an extended DDM (Ratcliff et al. 2016) with across-trial variability in drift rate. However, such a model is more challenging to fit to behavior data.
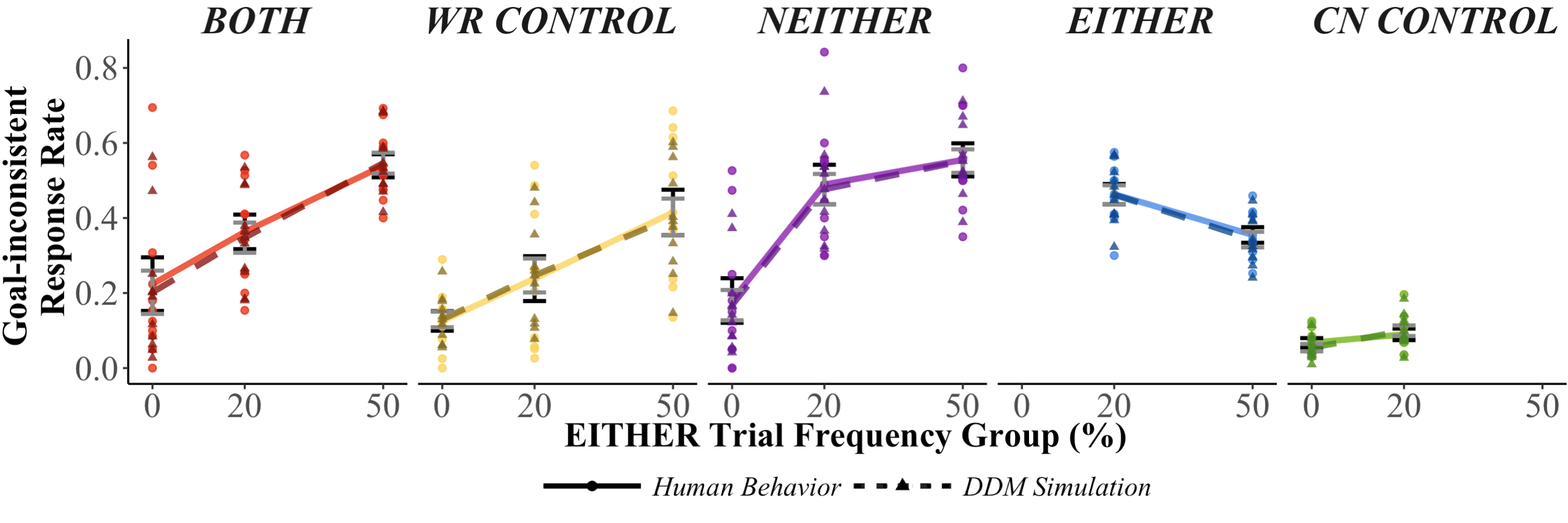


**Supplementary Figure 2:** *Simulated DDM goal-inconsistent response rate comparison to human behavior.* Solid lines and points are behavioral data. Dotted lines and triangles are simulated data. Black error bars indicate standard error of the mean for behavioral data. Grey error bars indicate standard error of the mean for simulated data. DDM simulation captures all of the goal-inconsistent response rate patterns.**
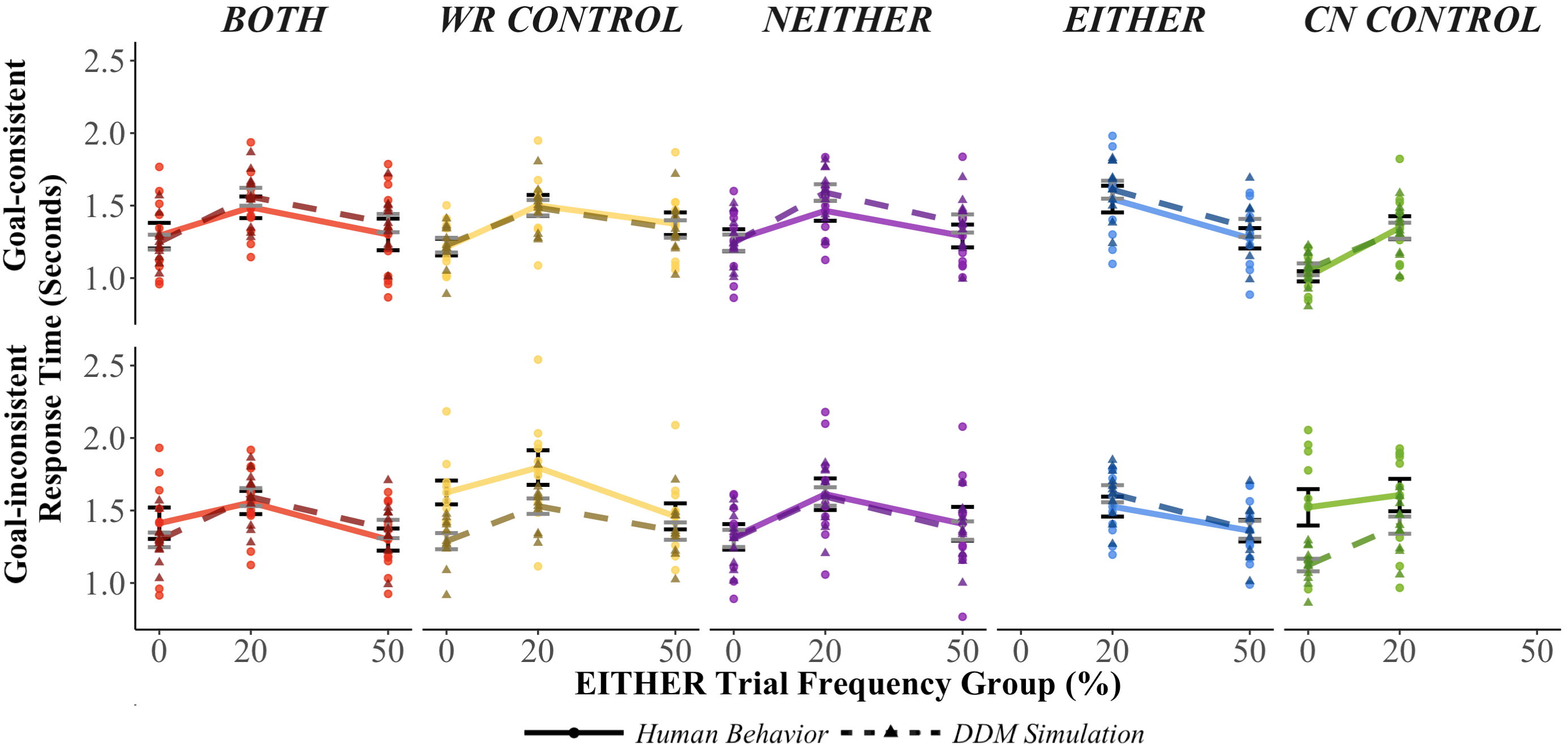
**

**Supplementary Figure 3:** *Simulated DDM response times comparison to human behavior.* Top, goal-consistent trials. Bottom, goal-inconsistent trials. Solid lines and points are behavioral data. Dotted lines and triangles are simulated data. Black error bars indicate standard error of the mean for behavioral data. Grey error bars indicate standard error of the mean for simulated data. DDM simulation captured many of the response time patterns, but produced similar patterns for goal-consistent and goal-inconsistent trials, whereas participants were slower on goal-inconsistent versus goal-consistent trials for many trial types.

**Bayesian analysis**

We conducted a Bayesian analysis to quantify exactly how confident we can be about each of the positive and negative findings. The advantage of this approach is able to distinguish between inconclusive evidence (for example, equal likelihood of the null and alternative) and evidence for the null hypothesis (e.g. higher likelihood of the null model) (Wagenmakers et al., 2010). We computed a Bayes Factor, which is ratio, based on a comparison of models, of how likely the null hypothesis is compared to the alternative hypothesis (Jarosz & Wiley, 2014). Bayes Factors can be thought of as the weight of evidence coming from the data. To do so we first performed Bayesian regression (package rstanarm, R programming language). We used the same procedure for the Bayesian regressions as in the mixed-effects regressions described in the Methods section (for each trial type, we predicted goal-inconsistent responses by an intercept and parameter for the percent of EITHER trials, with random effect of intercept by participant). We used a wide prior for both the intercept and beta value ($N(0,5)$). For each trial type we drew 4,000 samples (2,000 sample burn-in). All models converged. The pattern of results was the same as the frequentist regression, and the 95% highest density intervals do not overlap with zero for all parameters that were identified as significant by the frequentist regression (Figure S4).
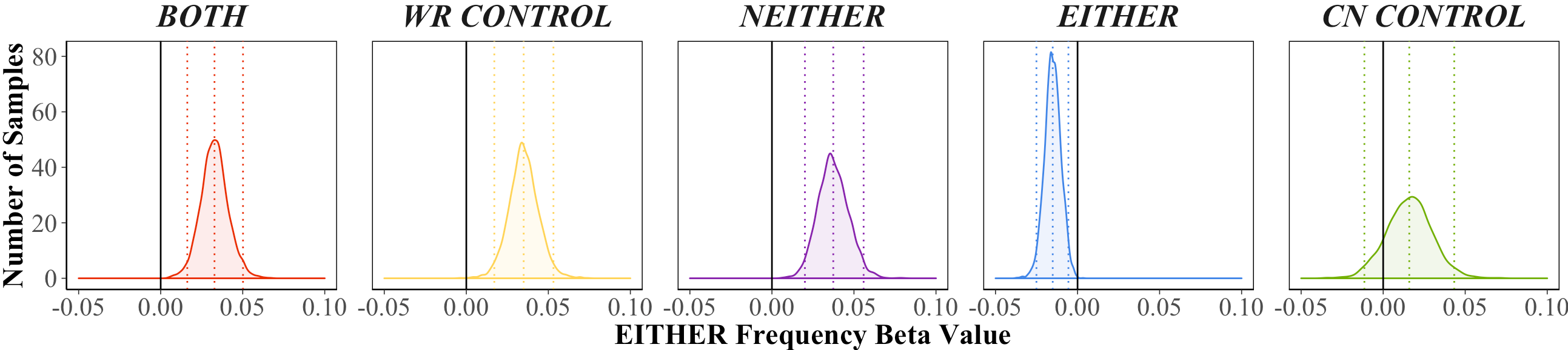


**Supplementary Figure 4.** Posterior distributions of sampled values of the effect of percent EITHER trials on goal-inconsistent response rate, for each trial type. Dashed vertical lines represent the mean of the distribution and the 95% highest density interval.

Then, we computed the Bayes Factor of the Bayesian regression models using the Savage-Dickey density ratio method, which represents the evidence *against* the null effect model (Wagenmakers et al., 2010; using bayestestR package in the R programming language). A Bayes Factor above 3 represents “substantial” evidence for the alternative hypothesis (Jarosz & Wiley, 2014). We see that the Bayes Factor hypothesis testing results in the same conclusions as we reached via the frequentist regressions for all of the significant results. In the case of the one non-significant result, the Bayes Factor can be inverted to represent evidence for the null effect model, which leads us to conclude there is substantial evidence for the null. As such, the number of data points in the present experiment was sufficient to yield substantial, strong, or decisive evidence for or against every single one of the effects according to the standard interpretation of Bayes factors introduced by Kass and Raftery (1995).

| **Bayesian regression analysis** | **Bayes factor** | **Inverse Bayes factor** |
| --- | --- | --- |
| % EITHER trials on the frequency of goal-inconsistent responses | 775300 |  |
| % EITHER trials on goal-inconsistent responses on BOTH trials | 15.663 |  |
| % EITHER trials on goal-inconsistent responses on NEITHER trials | 29.115 |  |
| % EITHER trials on goal-inconsistent responses on EITHER trials | 4.069 |  |
| % EITHER trials on goal-inconsistent responses on CN Control trials | 0.118 | 8.475 |
| % EITHER trials on goal-inconsistent responses on WR Control trials | 10.274 |  |

**Supplementary Table 3.** Bayes factors, reflecting the weight of the evidence *against* the null hypothesis, for each experimental effect. Inverse Bayes factor reflects the weight of the evidence *in favor of* the null hypotheses.

**Instructions**

Below are the exact instructions that were read aloud to participants.

[Screen 1]

“You will now practice the color associations for each button. You will see a series of colored X’s and simply need to press the key associated with the COLOR of those X’s.

For your left hand,

If the color is ‘orange’ press the 1st button,

if the color is ‘white’ press the 2nd button,

if the color is ‘green’ press the 3rd button,

and if the color is ‘yellow’ press the 4th button.

For your right hand,

If the color is ‘yellow’ press the 5th button,

if the color is ‘blue’ press the 6th button,

if the color is ‘brown’ press the 7th button,

and if the color is ‘pink’ press the 8th button.

We will let you know after each trial if you answered correctly or incorrectly.

Press any key to continue…”

[Screen 2, experimenter asks participant to say the word, and color, aloud]

“In the next task you will see a series of words written in colored ink, you may respond to either the word or the color.

RED

Press any key to continue…”

[Screen 3]

“You will receive rewards for being accurate on certain types of trials. If you are correct, the value of the trial will be displayed with a plus sign and in bold (e.g., +2). Points displayed in bold will be added to your total reward. If you are incorrect, you get zero points but still see the number of points you could have won, displayed without a plus sign (e.g., 2). Press any key to begin…”

[Screen 4, begin Mapping Phase Part 2]

“You will complete 3 blocks of trials, points you earn in each block will be displayed at the end of the block, at which time you can take a break. 200 points equals one dollar of bonus payment. Try to respond as quickly and accurately as possible. You will have 3 seconds to respond to each word. Press any key to begin…”

[Screen 5, Mapping Phase Part 2]

“You earned [N] points since last break. Take a break.

Press any key to begin the next part…”

[Screen 6, begin Transfer Phase]

“You earned [N] points since last break. Take a break.

Press any key to advance…”

[Screen 7, end]

“You’ve finished the experiment, you earned $[N]. Please notify the experimenter.”
